# Genomewide identification and analysis of heat‐shock proteins 70/110 to reveal their potential functions in Chinese soft‐shelled turtle *Pelodiscus sinensis*

**DOI:** 10.1002/ece3.5264

**Published:** 2019-05-20

**Authors:** Tengfei Liu, Yawen Han, Ye Liu, Huiying Zhao

**Affiliations:** ^1^ College of Veterinary Medicine Northwest A&F University Yangling China

**Keywords:** Chinese soft‐shelled turtle *Pelodiscus sinensis*, expression profiling, heat‐shock proteins 70/110, protein interaction, regulation of apoptosis

## Abstract

Heat‐shock proteins 70/110 (Hsp70/110) are vital molecular chaperones and stress proteins whose expression and production are generally induced by extreme temperatures or external stresses. The Hsp70/110 family is largely conserved in diverse animals. Although many reports have studied and elaborated on the characteristics of Hsp70/110 in various species, the systematic identification and analysis of Hsp70/110 are still poor in turtles. In this study, a genomewide search was performed, and 18 candidate *PsHSP70/110* family genes were identified in Chinese soft‐shelled turtle, *Pelodiscus sinensis*. These PsHSP70/110 proteins contained the conserved “heat shock protein 70” domain. Phylogenetic analysis of PsHSP70/110 and their homologs revealed evolutionary conservation of Hsp70/110 across different species. Tissue‐specific expression analysis showed that these *PsHSP70/110* genes were differentially expressed in different tissues of *P. sinensis*. Furthermore, to examine the putative biological functions of *PsHSP70/110*, the dynamic expression of *PsHSP70/110* genes was analyzed in the testis of *P. sinensis* during seasonal spermatogenesis following germ cell apoptosis. Notably, genes such as *PsHSPA1B‐L*, *PsHSPA2*, and *PsHSPA8* were significantly upregulated in *P. sinensis* testes along with a seasonal decrease in apoptosis. Protein interaction prediction revealed that PsHSPA1B‐L, PsHSPA2, and PsHSPA8 may interact with each other and participate in the MAPK signaling pathway. Moreover, immunohistochemical analysis showed that PsHSPA1B‐L, PsHSPA2, and PsHSPA8 protein expression was associated with seasonal temperature variation. The expression profiling and interaction relationships of the PsHSPA1B‐L, PsHSPA2, and PsHSPA8 proteins implied their potential roles in inhibiting the apoptosis of germ cells in *P. sinensis*. These results provide insights into PsHSP70/110 functions and will serve as a rich resource for further investigation of HSP70/110 family genes in *P. sinensis* and other turtles.

## 1 INTRODUCTION

Heat‐shock proteins (Hsps), which were first reported in *Drosophila*, are ubiquitously found in bacteria, plants, and animals (Arya, Mallik, & Lakhotia, 2007). When organisms are exposed to extreme temperatures or external stresses such as disease, toxins, and hypoxia, Hsps can be synthesized as stress proteins and accumulate to respond to various environmental insults (Gupta, Sharma, Mishra, Mishra, & Chowdhuri, 2010; Srivastava, 2002). Based on the approximate molecular weight and amino acid sequence homologies of Hsps, they are distinctly classified into five major families: Hsp110, Hsp90, Hsp70, Hsp60, and small Hsps (sHsps) (Lindquist & Craig, 2003). Among these proteins, Hsp70 is one of the most heat inducible and evolutionally conserved in terms of structure and function (Murphy, 2013). Hsp70 proteins generally form two groups: stress‐inducible Hsp70 proteins and constitutively expressed heat‐shock cognate 70 (Hsc70) proteins, according to expression profiling (Kiang & Tsokos, 1998). Two major conserved domains, a nucleotide‐binding domain (NBD) and a substrate‐binding domain (SBD), are characteristic of Hsp70 family proteins (Bertelsen, Chang, Gestwicki, & Zuiderweg, 2009). The intrinsic activities and allosteric coupling of NBD and SBD are associated with the functions of Hsp70 (Bertelsen et al., 2009). Specifically, Hsp110, which exhibits a longer C‐terminal extension, shares the same domain organization and exhibits highly similar crystal structures to Hsp70, which reveals the close relationships of the Hsp110 and Hsp70 protein families (Dragovic, Broadley, Shomura, Bracher, & Hartl, 2006).

By functioning as a molecular chaperone in the folding, denaturation, degradation, and inhibition of proteins and controlling regulatory proteins, Hsp70 plays essential roles in heat adaptation and protection against stresses in diverse species (Murphy, 2013). Extensive evidence has suggested that Hsp70 not only exhibits ATP‐dependent chaperoning function but is also a negative apoptosis‐inducing factor (AIF) in response to a wide range of stimuli (Goloudina, Demidov, & Garrido, 2012; Jiang et al., 2011; Sabirzhanov, Stoica, Hanscom, Piao, & Faden, 2012). In rodent models, overexpression of Hsp70 provides a survival advantage to tumor cells because Hsp70 can interact with multiple components of the apoptotic machinery (Jäättelä, 1995). Conversely, it has been reported that Hsp70 knockdown leads to decreased cell proliferation and facilitates the induction of apoptosis in multiple cancer cell models (Kotoglou et al., 2009; Zhang et al., 2013). Indeed, Hsp70 can block apoptosis by binding to apoptosis protease‐activating factor 1 (Apaf1), thereby preventing the recruitment of procaspase‐9 to the apoptosome (Beere et al., 2000). Similarly, Hsp70 regulates the important apoptotic mediator Bax and prevents Bax from translocating to mitochondria, which is necessary for the disruption of the mitochondrial membrane (Stankiewicz, Lachapelle, Foo, Radicioni, & Mosser, 2005). The regulatory roles of Hsp70 in apoptosis are also due to the effects of Hsp70 on stress‐induced kinases, including SAPK/JNK, p38, and apoptosis signal‐regulating kinase (Park et al., 2002; Park, Lee, Huh, Seo, & Choi, 2001). Additionally, Hsp70 can inhibit caspase‐independent apoptosis by directly interacting with AIF and cathepsins (Jesper et al., 2004; Ravagnan et al., 2001). Despite considerable research advances, the antiapoptotic mechanism of Hsp70 is still controversial, especially in nonmodel animals. In recent studies, many genes encoding Hsp70 have been identified and characterized from nonmodel animals such as amphibians, insects, crustaceans, mollusks, and fishes (Luan et al., 2010; Simoncelli, Morosi, Rosa, Pascolini, & Fagotti, 2010; Song et al., 2016; Wang et al., 2019; Wang, Wu, Jian, & Lu, 2009), enriching knowledge of the phylogenetic relationships and biological functions of Hsp70. However, few studies have focused on the genomewide identification and functional analysis of the Hsp70 gene family in turtles.

Chinese soft‐shelled turtle (*Pelodiscus sinensis*), a reptile, presents important economic value and is widely distributed in Asian countries such as China, Japan, and Korea. *P. sinensis* is an ectothermic aquaculture species with a specific evolutionary role linking ectothermic anamniotic animals (fishes and amphibians) and endothermic amniotic animals (birds and mammals) (Zimmerman, Vogel, & Bowden, 2010) and can thus be used as a potential animal model to study the evolution of critical genes or species (Liu, Chu, et al., 2016). The body temperature of *P. sinensis* is dependent on the ambient temperature, similar to other ectotherms, resulting in typical hibernation patterns in midwinter (Chen et al., 2015). Previous studies have revealed distinct seasonal apoptosis in the testis of *P. sinensis* on the basis of morphological and molecular evidence (Liu et al., 2017). Furthermore, it is well known that Hsp70/110 presents the obligatory function of responding to adverse external stimuli, especially heat shock, and exhibits survival‐promoting effects and suppression of apoptosis (Gao et al., 2014). However, the relationship between apoptosis and stress‐related Hsp70/110 is poorly understood in *P. sinensis*. Fortunately, the public genomic sequences and RNA‐seq data of *P. sinensis* (Liu et al., 2017; Liu, Yang, et al., 2016; Wang et al.,^2013^) can provide rich resources for the identification, phylogenetic analysis and functional exploration of PsHsp70/110 genes in *P. sinensis*.

The primary goals of this study were to systematically identify candidate *PsHSP70/110* family genes based on the whole‐genome sequence of *P. sinensis* and to analyze their classification, conserved structures, and phylogenetic relationships. Furthermore, we focused on the mRNA and protein expression of *PsHSP70/110* genes to investigate their putative roles in germ cell apoptosis following seasonal temperature variation. These results will provide useful information for the exploration of PsHSP70/110 functions in *P. sinensis* and facilitate further investigation of Hsp70/110 family genes in turtles.

## 2 MATERIALS AND METHODS

### 2.1 Animals

In this study, all sample procedures and animal care were conducted according to the guidelines of the Animal Research Institute Committee (Northwest A&F University, Shaanxi, China). The protocol was approved by the Science and Technology Agency of Shaanxi Province under permit NO. SYXK (SN) 2018‐0003. All efforts were made to minimize animal suffering. Healthy adult Chinese soft‐shelled turtles (3–4 years old with an average weight of 1.09 ± 0.15 kg) were captured from Yangcheng Lake in Suzhou (31°N, 120°E), Jiangsu province, China. Suzhou has a typical temperate climate with four distinct seasons, including spring (March–May), summer (June–August), autumn (September–November), and winter (December–February). Prior to the experiments, the turtles were acclimated in a tank with a recirculating water system for one week. To analyze the tissue‐specific expression of *PsHSP70/110* mRNA in *P. sinensis*, five male and five female turtles that were breeding at 25 ℃ were anaesthetized by intraperitoneal administration of sodium pentobarbital (20 mg/kg) and killed by cervical dislocation. Tissue samples (including muscle, heart, blood, brain, kidney, liver, lung, spleen, intestine, testis, and oviduct) were collected immediately. For dynamic expression analysis of *PsHSP70/110* genes in the testis, male turtles collected in April (spring, average temperature 15.7℃), July (summer, average temperature 28.6℃), and October (autumn, average temperature 18.4℃) in 2017 were used to obtain testis samples with five replicates. The testis sample from one side of each turtle was fixed for immunohistochemistry (IHC) analysis. The other side of the testis was placed in liquid nitrogen immediately and kept at −80℃ until RNA extraction.

### 2.2 Identification and conserved domains of *Hsp70/110* family genes in *P. sinensis*

The public *P. sinensis* genome sequences (Wang et al., 2013) were used to identify potential *Hsp70/110* genes via genomewide searches. A BLASTp homology search against the NCBI database was also performed by using the reported Hsp70/110 sequences from human and mouse as a query. To confirm the candidate *PsHSP70/110* genes, the obtained protein sequences were further subjected to analysis of conserved domains against public databases including NCBI Conserved Domain Database (http://www.ncbi.nlm.nih.gov/cdd), Pfam (http://pfam.xfam.org), and InterProScan (http://www.ebi.ac.uk/Tools/pfa/iprscan5). The nucleotide and amino acid sequences of *PsHSP70/110* genes were retrieved and used for further analysis. These candidate *PsHSP70/110* family genes were named according to the homologous gene names, annotations, and gene descriptions in the NCBI database. The physical and chemical characteristics of *PsHSP70/110* genes were analyzed by using ExPASy (http://web.expasy.org/protparam). The molecular weights, theoretical pI, instability index, aliphatic index, and grand average of hydropathicity (GRAVY) were calculated.

### 2.3 Phylogenetic analysis and alignment of *Hsp70/110* genes in different species

To detect the phylogenetic relationship of *PsHSP70/110* genes, the protein sequences of *Hsp70/110* family genes from humans (*Homo sapiens*), house mouse (*Mus musculus*), platypus (*Ornithorhynchus anatinus*), turkey (*Meleagris gallopavo*), chicken (*Gallus gallus*), American alligator (*Alligator mississippiensis*), green anole (*Anolis carolinensis*), western painted turtle (*Chrysemys picta bellii*), three‐toed box turtle (*Terrapene mexicana triunguis*), green sea turtle (*Chelonia mydas*), African clawed frog (*Xenopus laevis*), and zebrafish (*Danio rerio*) were downloaded from the NCBI database. The phylogenetic relationships among different species were analyzed using the TimeTree server (http://www.timetree.org). The full‐length sequences of Hsp70/110 proteins from different species were used for sequence alignment and phylogenetic analysis. Sequence alignment was performed by using MAFFT software. The phylogenetic tree was constructed by using PhyML 3.0 software (Guindon et al., 2010) based on the maximum‐likelihood (ML) method and bootstrap values with 1,000 replications.

### 2.4 Quantitative real‐time PCR analysis

Quantitative real‐time PCR (qRT‐PCR) analysis was performed according to previous reports (Liu et al., 2017; Liu, Yang, et al., 2016). Total RNA was extracted using TRIzol reagent (Life Technologies). cDNA was synthesized using the SuperScript First‐Strand Synthesis System (Invitrogen). Primer sequences for gene expression analysis were designed using Beacon Designer software (Premier Biosoft International). The relative expression levels of genes were normalized to *β*‐actin and analyzed using the 2^−ΔΔ^ *^C^* ^T ^method (Livak & Schmittgen, 2001).

### 2.5 Transcriptomic expression analysis of *PsHSP70/110* genes in *P. sinensis*

The dynamic expression patterns of *PsHSP70/110* genes in the testis of *P. sinensis* in different months were detected by using published RNA‐seq data from *P. sinensis* testis to calculate gene expression levels. Transcriptomic analysis of *P. sinensis* testes in April (spermatogenically quiescent phase, MT‐1), July (intermediate spermatogenesis, MT‐2), and October (late spermatogenesis, MT‐3) was performed in our previous study (Liu et al., 2017). RNA‐seq data from the three libraries are available in the NCBI Sequence Read Archive (SRA, http://www.ncbi.nlm.nih.gov/Traces/sra) under accession numbers SRX2351846 (MT‐1), SRX2352135 (MT‐2), and SRX2352136 (MT‐3). The expression patterns of *PsHSP70/110* genes in different months were calculated by the fragments per kb per million reads (FPKM) method (Mortazavi, Williams, McCue, Schaeffer, & Wold, 2008). The FPKM value associated with an abundance of zero was set to 0.01 for gene expression analysis. Heat maps of gene expression were generated by using Cluster software (de Hoon, Imoto, Nolan, & Miyano, 2004) and Java Treeview software (Saldanha, 2004).

### 2.6 Interaction network and functional enrichment of PsHSP70/110 proteins

The protein sequences of *PsHSP70/110* genes were used for analyzing protein–protein associations. The interaction relationships among PsHSP70/110 genes from *P. sinensis* were determined by using online STRING software (http://string-db.org). The interaction networks were constructed by setting the minimum required interaction score to ≥0.7. The functional enrichments (*P*‐value ≤ 0.05) in the interaction network were analyzed based on the Kyoto Encyclopedia of Genes and Genomes (KEGG, http://www.kegg.jp/kegg/pathway.html), Pfam, and InterProScan databases.

### 2.7 Immunohistochemistry

Immunohistochemistry was performed as previously described (Liu et al., 2017). Briefly, sections of *P. sinensis* testis were dewaxed in xylene, dehydrated in an ethanol series, and blocked with 3% H_2_O_2_ in distilled water. The sections were incubated with anti‐HSPA1L/HSPA1B‐L (ab154409, Abcam Inc., Cambridge, MA, USA; 1:100), anti‐HSPA2 (ab154374, Abcam Inc., Cambridge, MA, USA; 1:100), and anti‐Hsc70/HSPA8 (ab19136, Abcam Inc.; 1:150) antibodies separately overnight at 4℃. Negative controls were reacted with PBS instead of the specific antibodies. A biotinylated secondary antibody and Vector ABC reagent (Vector Laboratories) were subsequently added according to the manufacturer's instructions. Then, the sections were stained using the FAST DAB Peroxidase Substrate (Sigma) and counterstained with hematoxylin for 10 s. The slides were dehydrated and analyzed under a light microscope.

### 2.8 Prediction of 3D protein structures

The 3D structures of PsHSP70/110 proteins were predicted for further analysis of protein functions. The 3D protein structures were generated by a homology modeling method using the known homologous structure as a template. The Phyre2 server (Kelley, Mezulis, Yates, Wass, & Sternberg, 2015) was used for homology modeling, secondary structure prediction, and domain analysis. The comparison of 3D protein structures was performed by using PyMOL Viewer software. The prediction of binding sites in proteins was performed using similar structures on the 3DLigandSite server (Wass, Kelley, & Sternberg, 2010).

### 2.9 Data analysis

The data were expressed as the means ± *SEM*. One‐way ANOVA was performed using SPSS 16.0 software to assess the differences in gene expression levels. The level of significance was set at a *p*‐value < 0.05.

## 3 RESULTS

### 3.1 Identification and analysis of *Hsp70/110* genes in *P. sinensis*

**Figure 1:**
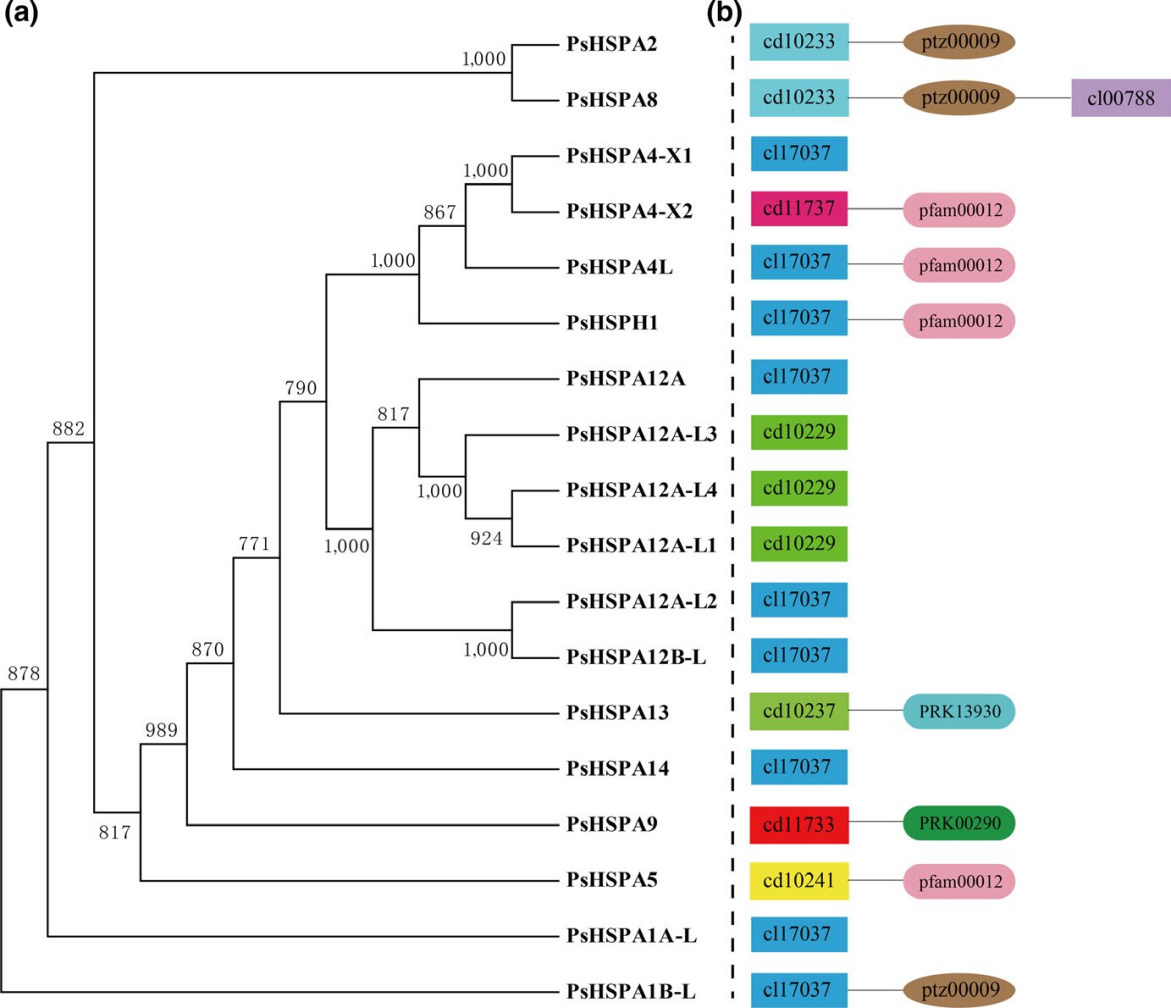
Characterization of the identified *PsHSP70/110* genes in *Pelodiscus sinensis*. (a) Phylogenetic analysis of PsHSP70/110 proteins using bootstrap values with 1,000 replications. (b) A diagram of conserved domains in PsHSP70/110 proteins

In this study, a genomewide search against the *P. sinensis* genome sequences generated 18 candidate genes belonging to the *HSP70/110* family, including 17 *Hsp70* genes and one *Hsp110* gene (Table 1), and these genes were considered as the *PsHSP70/110* family genes of *P. sinensis*. PsHSP70/110 protein sequences were obtained and used for phylogenetic analysis. According to the genetic distances among the 18 PsHSP70/110 proteins, they were classified into different branches (Figure 1a). Moreover, an analysis of conserved domains showed that all the PsHSP70/110 proteins contained an NBD domain (Table 1; Figure 1b), which was in accord with the typical characteristic of Hsp70/110 proteins. Notably, half of these PsHSP70/110 proteins, such as PsHSPA1A‐L, PsHSPA1B‐L, PsHSPA12A, and PsHSPA14, exhibited an NBD_sugar‐kinase_HSP70_actin (cl17037) domain.

**Table 1:** The identification and conserved domains of *PsHSP70/110* genes in *Pelodiscus sinensis*

Gene name	NCBI accession	Conserved domains
PsHSPA1A‐L	XM_014572517.1, XP_014428003.1	cl17037, NBD_sugar‐kinase_HSP70_actin
PsHSPA1B‐L	XM_006134687.2, XP_006134749.1	cl17037, NBD_sugar‐kinase_HSP70_actin; PTZ00009, heat‐shock 70‐kDa protein
PsHSPA2	NM_001287561.1, NP_001274490.1	cd10233, HSPA1−2_6−8‐like_NBD; PTZ00009, heat‐shock 70‐kDa protein
PsHSPA4‐X1	XM_006128531.2, XP_006128593.1	cl17037, NBD_sugar‐kinase_HSP70_actin
PsHSPA4‐X2	XM_006128532.2, XP_006128594.1	cd11737, HSPA4_NBD; pfam00012, Hsp70 protein
PsHSPA4L	XM_014577892.1, XP_014433378.1	cl17037, NBD_sugar‐kinase_HSP70_actin; pfam00012, Hsp70 protein
PsHSPA5	NM_001286892.1, NP_001273821.1	cd10241, HSPA5‐like_NBD; pfam00012, HSP70
PsHSPA8^a^	NM_001286908.1, NP_001273837.1	cd10233, HSPA1−2_6−8‐like_NBD; PTZ00009, heat‐shock 70‐kDa protein; cl00788, MttA_Hcf106
PsHSPA9	XM_014569866.1, XP_014425352.1	cd11733, HSPA9‐like_NBD; PRK00290, molecular chaperone DnaK
PsHSPA12A	XM_006120999.2, XP_006121061.1	cl17037, NBD_sugar‐kinase_HSP70_actin
PsHSPA12A‐L1	XM_006127821.2, XP_006127883.1	cd10229, HSPA12_like_NBD
PsHSPA12A‐L2	XM_014573716.1, XP_014429202.1	cl17037, NBD_sugar‐kinase_HSP70_actin
PsHSPA12A‐L3	XM_014572222.1, XP_014427708.1	cd10229, HSPA12_like_NBD
PsHSPA12A‐L4	XM_006127819.2, XP_006127881.1	cd10229, HSPA12_like_NBD
PsHSPA12B‐L	XM_014570751.1, XP_014426237.1	cl17037, NBD_sugar‐kinase_HSP70_actin
PsHSPA13	XM_006136928.2, XP_006136990.1	cd10237, HSPA13‐like_NBD; PRK13930, rod shape‐determining protein MreB
PsHSPA14	XM_014570505.1, XP_014425991.1	cl17037, NBD_sugar‐kinase_HSP70_actin
PsHSPH1	XM_006125320.1, XP_006125382.1	cl17037, NBD_sugar‐kinase_HSP70_actin; pfam00012, HSP70

a Also known as PsHsc70.

The analysis of amino acid identity showed that members of the same PsHSP70/110 subfamily shared higher sequence similarities and similar domain structures (Figure 1; Table 2). For example, PsHSPA2 and PsHSPA8 shared 89% amino acid identity with each other, and both contained the HSPA1‐2_6‐8‐like_NBD (cd10233) and heat‐shock 70‐kDa protein (PTZ00009) domains. In addition, PsHSPH1, belonging to the HSP110 proteins, exhibited lower sequence identities with PsHSP70 proteins (less than 40%) with the exceptions of PsHSPA4L, PsHSPA4‐X1, and PsHSPA4‐X2 (Table 2). Furthermore, analyses of physical and chemical characteristics showed that the molecular weights of most PsHSP70/110 proteins ranged from 50.3 to 77.3 kDa, with the exceptions of PsHSPA4‐X1 (94.4 kDa) and PsHSPA12A‐L2 (37.2 kDa) (Table S1). The theoretical pI values of most proteins ranged from 5.01 to 5.95, with the exception of PsHSPA12A (7.64). All detailed information on PsHSP70/110 proteins, including their instability index, aliphatic index, and GRAVY, is listed in Table S1.

**Table 2:** The sequence identity of PsHSP70/110 proteins in *Pelodiscus sinensis* compared with each other

Amino acid identity	PsHSPA1A‐L (%)	PsHSPA1B‐L (%)	PsHSPA2 (%)	PsHSPA4L (%)	PsHSPA4‐X1 (%)	PsHSPA4‐X2 (%)	PsHSPA5 (%)	PsHSPA8 (%)	PsHSPA9 (%)	PsHSPA12A (%)	PsHSPA12A‐L1 (%)	PsHSPA12A‐L2 (%)	PsHSPA12A‐L3 (%)	PsHSPA12A‐L4 (%)	PsHSPA12B‐L (%)	PsHSPA13 (%)	PsHSPA14 (%)	PsHSPH1 (%)
PsHSPA1A‐L	100.0																	
PsHSPA1B‐L	55.0	100.0																
PsHSPA2	58.0	61.0	100.0															
PsHSPA4L	18.6	32.0	22.2	100.0														
PsHSPA4‐X1	20.9	20.0	33.0	64.0	100.0													
PsHSPA4‐X2	14.9	19.9	30.0	63.0	81.9	100.0												
PsHSPA5	49.0	52.0	63.0	21.3	25.3	15.4	100.0											
PsHSPA8	59.0	61.0	89.0	32.0	33.0	31.0	66.0	100.0										
PsHSPA9	40.4	49.0	54.0	21.4	25.6	16.9	54.0	53.0	100.0									
PsHSPA12A	14.5	16.1	16.6	10.8	12.1	13.1	16.6	16.5	60.0	100.0								
PsHSPA12A‐L1	16.1	14.6	15.1	11.7	12.3	16.5	15.8	15.2	15.9	31.0	100.0							
PsHSPA12A‐L2	12.8	15.2	12.0	8.7	9.6	12.6	12.8	11.5	12.0	15.8	14.6	100.0						
PsHSPA12A‐L3	16.9	14.6	17.4	11.1	13.3	14.6	13.6	16.2	13.8	31.0	85.4	13.2	100.0					
PsHSPA12A‐L4	17.4	15.9	16.4	10.7	13.0	14.7	15.6	15.7	12.6	25.2	86.8	13.1	94.8	100.0				
PsHSPA12B‐L	13.3	14.3	12.5	9.1	8.8	11.2	11.7	12.1	12.4	65.0	43.0	38.2	41.0	17.0	100.0			
PsHSPA13	23.3	37.0	39.0	12.1	16.0	13.1	26.8	39.0	36.0	18.0	14.4	15.5	14.6	14.1	13.5	100.0		
PsHSPA14	26.3	33.0	34.0	17.4	17.8	14.4	33.0	34.0	25.2	15.8	17.0	15.3	16.1	16.4	15.1	22.1	100.0	
PsHSPH1	20.7	21.9	34.0	57.0	60.0	60.0	22.7	33.0	22.2	10.5	10.7	13.1	12.9	11.9	9.9	11.7	15.8	100.0

### 3.2 Phylogenetic analysis of *Hsp70/110* genes

**Figure 2:**
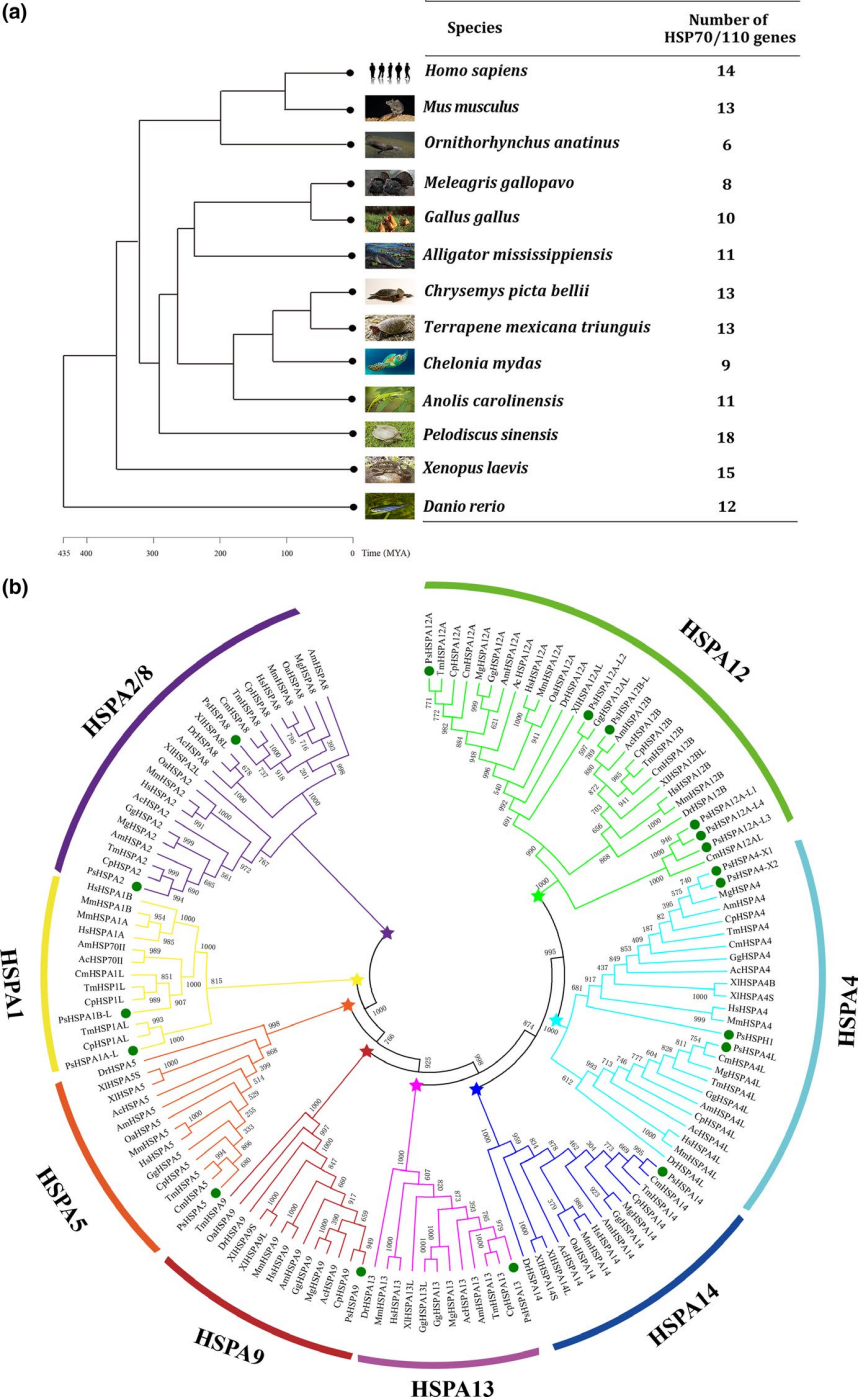
Overview of Hsp70/110 proteins in 13 species. (a) The number of *HSP70/110* genes in different species. MYA: million years ago. (b) Phylogenetic relationships and classification of HSP70/110 homologous proteins using bootstrap values with 1,000 replications. The green circle indicates the PsHSP70/110 proteins in *Pelodiscus sinensis*. Asterisks in different colors indicate the different subfamilies by their classification. Ac, *Anolis carolinensis*; Am, *Alligator mississippiensis*; Cm, *Chelonia mydas*; Cp, *Chrysemys picta bellii*; Dr, *Danio rerio*; Gg, *Gallus gallus*; Hs, *Homo sapiens*; Mg, *Meleagris gallopavo*; Mm, *Mus musculus*; Oa, *Ornithorhynchus anatinus*; Tm, *Terrapene mexicana triunguis*; Xl, *Xenopus laevis*

The homologous *Hsp70/110* genes from 12 other species were collected for comparative analysis with the identified *PsHSP70/110* genes (Figure 2). The statistics of the *Hsp70/110* genes revealed that most species harbored more than 10 *Hsp70/110* members, although platypus (*O. anatinus*), turkey (*M. gallopavo*), and green sea turtle (*C. mydas*) exhibited six, eight, and nine members, respectively (Figure 2a). More gene members of the *Hsp70/110* family were identified in *P. sinensis* than in other species. The protein sequences of 18 *PsHSP70/110* genes from *P. sinensis* and 124 homologous genes were used for constructing a phylogenetic tree (Table S2; Figure 2b). The results showed that the 142 Hsp70/110 proteins were grouped into eight distinct clades. The greatest number of Hsp70/110 genes was allocated to the HSPA12 subfamily, which was composed of HSPA12A, HSPA12B, and their corresponding homologous genes. The lists of PsHSPA4‐homologous genes constituted the second largest subfamily. Relatively, few genes belonged to the other subfamilies (Figure 2b). Notably, PsHSPA2 homologs shared the closest phylogenetic relationships with PsHSPA8 homologs, which was consistent with the high similarity between PsHSPA2 and PsHSPA8 in their amino acid sequences (Table 2). HSPA2 and HSPA8 genes were assigned to the same subfamily as HSPA2/8. Compared with the phylogenetic analysis of PsHSP70/110 shown in Figure 1a, the detailed classification shown in Figure 2b was performed to indicate the phylogenetic relationships among PsHSP70/110 genes and the homologous genes in other species.

### 3.3 Expression profiling of *PsHSP70/110* genes in different tissues of *P. sinensis*

**Figure 3:**
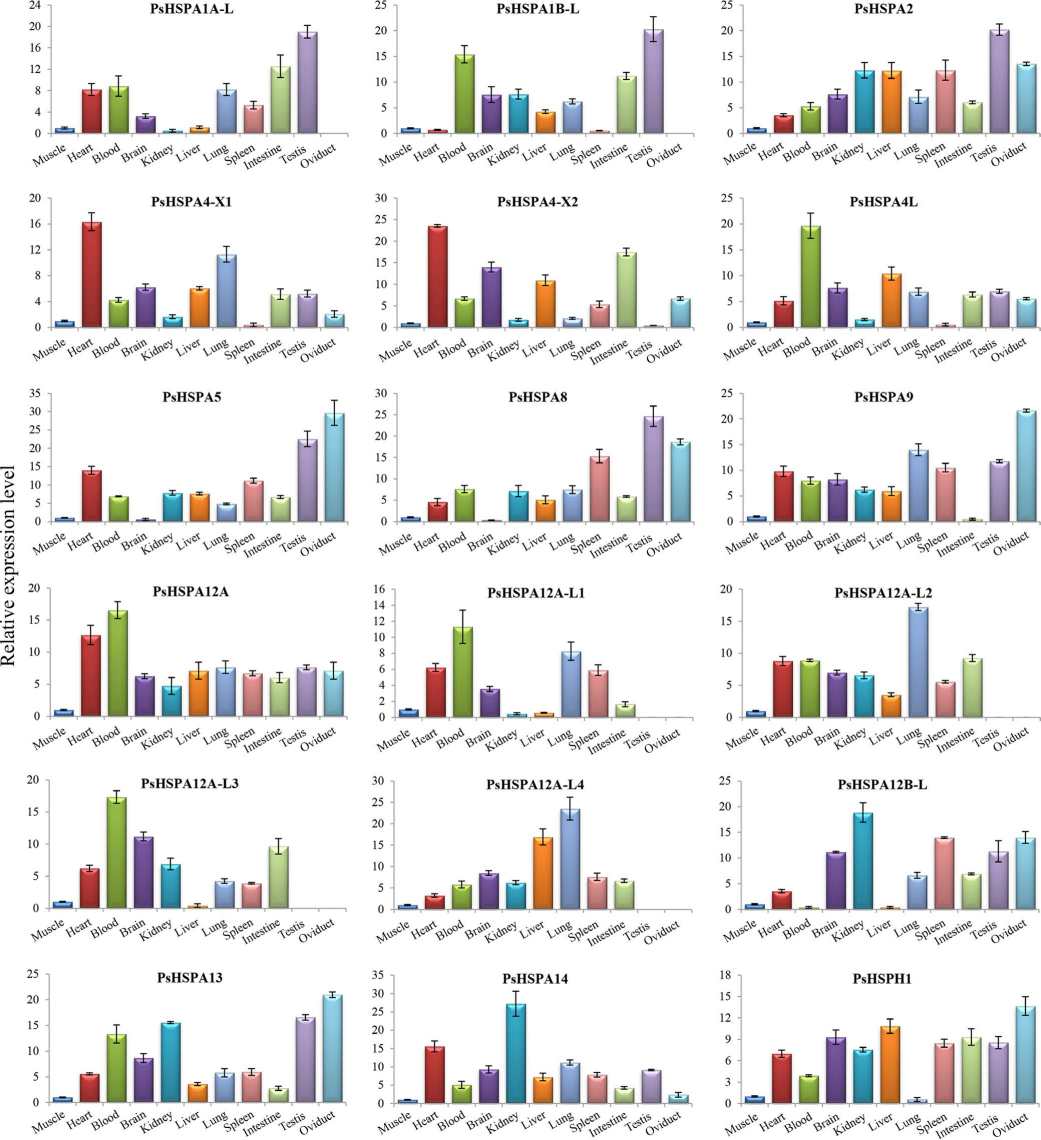
Expression patterns of *PsHSP70/110* genes in different tissues of *Pelodiscus sinensis*. Each bar shows the mean ± *SEM* of triplicate assays

Tissue‐specific expression analysis of *PsHSP70/110* genes by qRT‐PCR revealed that these *PsHSP70/110* genes showed differential expression patterns in different tissues (muscle, heart, blood, brain, kidney, liver, lung, spleen, intestine, testis, and oviduct) of *P. sinensis* (Figure 3; Table 3). *PsHSPA4‐X1* and *PsHSPA4‐X2* exhibited high expression levels in the heart, and *PsHSPA4L*, *PsHSPA12A*, *PsHSPA12A‐L1*, and *PsHSPA12A‐L3* were highly expressed in the blood. Relatively higher expression levels of *PsHSPA12B‐L* and *PsHSPA14* were detected in the kidney, and the highest levels of *PsHSPA12A‐L2* and *PsHSPA12A‐L4* were found in the lung. Importantly, *PsHSPA1A‐L*, *PsHSPA1B‐L*, *PsHSPA2*, and *PsHSPA8* were highly expressed in the testis, and *PsHSPA5*, *PsHSPA9*, *PsHSPA13*, and *PsHSPH1* were highly expressed in the oviduct. The results implied that these *PsHSP70/110* genes with specific high expression in reproductive organs (such as the testis and oviduct) may play roles in the reproductive development of *P. sinensis*. In addition, *PsHSPA12A‐L1*, *PsHSPA12A‐L2*, *PsHSPA12A‐L3*, and *PsHSPA12A‐L4* exhibited no expression in the testis and oviduct, and *PsHSPA1A‐L* and *PsHSPA1B‐L* were not expressed in the oviduct.

**Table 3:** The specific primers of *PsHSP70/110* genes for qRT‐PCR analysis

Gene name	Sense primer (5'−3')	Antisense primer (5'−3')
PsHSPA1A‐L	CTACAAGGGAAAGGAGAA	CATAGTCTTCAGCCATCT
PsHSPA1B‐L	TTGGCAGAGAAGGAGGAG	TGAGGTTGGAGAAGACAGA
PsHSPA2	TCTTGAGTCCTACACCTA	CACTTTCTGCTTGTCTTG
PsHSPA4‐X1	GCCTACTATACTTCTCCTAA	GCTCTTCATTCTCATCAG
PsHSPA4‐X2	GCCTACTATACTTCTCCTAA	GCTCTTCATTCTCATCAG
PsHSPA4L	TAAGAATGCTGTTGAAGAAT	CTGAATAGGCTGACCATA
PsHSPA5	GTAACAATCAAGGTCTAT	ATTCCATTCACATCTATC
PsHSPA8	CGTTGCCTTCACAGATAC	CGTGTTGGTAGGATTCATT
PsHSPA9	AATACATTCTACGCTACC	CTTCATCTTCATCAATACAA
PsHSPA12A	GAACCAATCCACTGAATA	CACTAAGATGTTGAATGAT
PsHSPA12A‐L1	ACTACTTCCACCACTTCAA	TTCCATTATCTGCGTCCA
PsHSPA12A‐L2	CTTGTTGTTGTGGCTATT	AATACATTCTGGCTCCTT
PsHSPA12A‐L3	ACAGTATCAGACGAACCAT	AAAGCCTCCAACCAGTAA
PsHSPA12A‐L4	AAGGAGTATGGGATGAATA	GGTTATGGTAGCAATGTAA
PsHSPA12B‐L	GGATTATTACCACGACCTC	CACCTTCTTCCCATTCAT
PsHSPA13	ACTCTACACCATTCTTCTC	TTCAACACCTGCTCAATA
PsHSPA14	AGAGCGATGATGAAGTTA	CCTGGACACATTACAATC
PsHSPH1	GAATGGATGAGTAATGCTA	ATTGTGTAGTTCCTTGAG
Psβ‐actin	AGACCCGACAGACTACCTCA	CACCTGACCATCAGGCAACT

### 3.4 Dynamic expression of *PsHSP70/110* genes during spermatogenesis in *P. sinensis*

**Figure 4:**
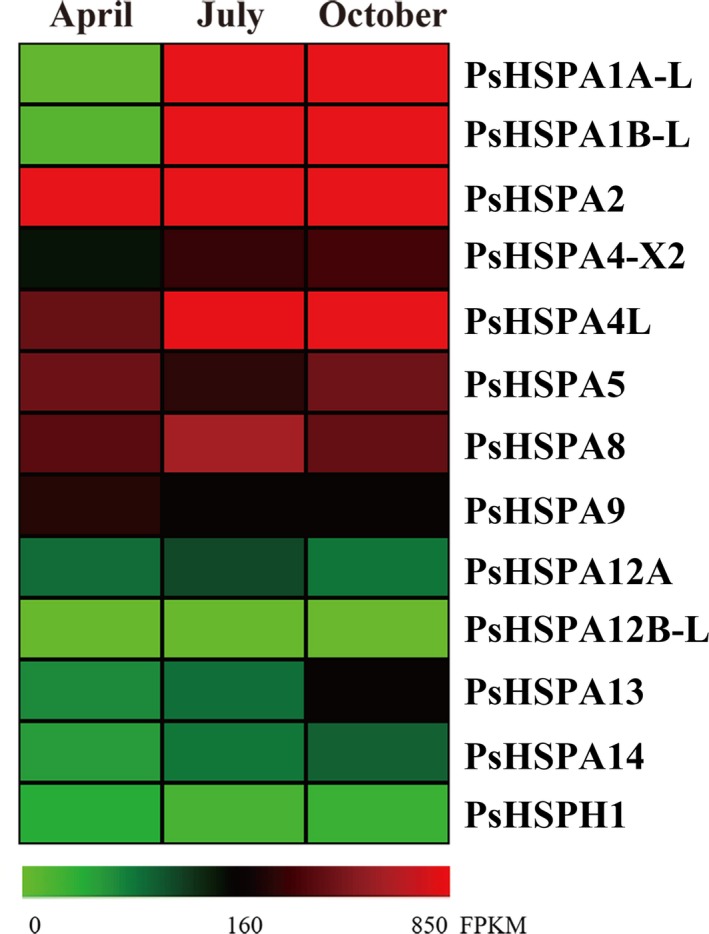
Heat map of *PsHSP70/110* gene expression in the testis of *Pelodiscus sinensis* based on RNA‐seq analysis

**Figure 5:**
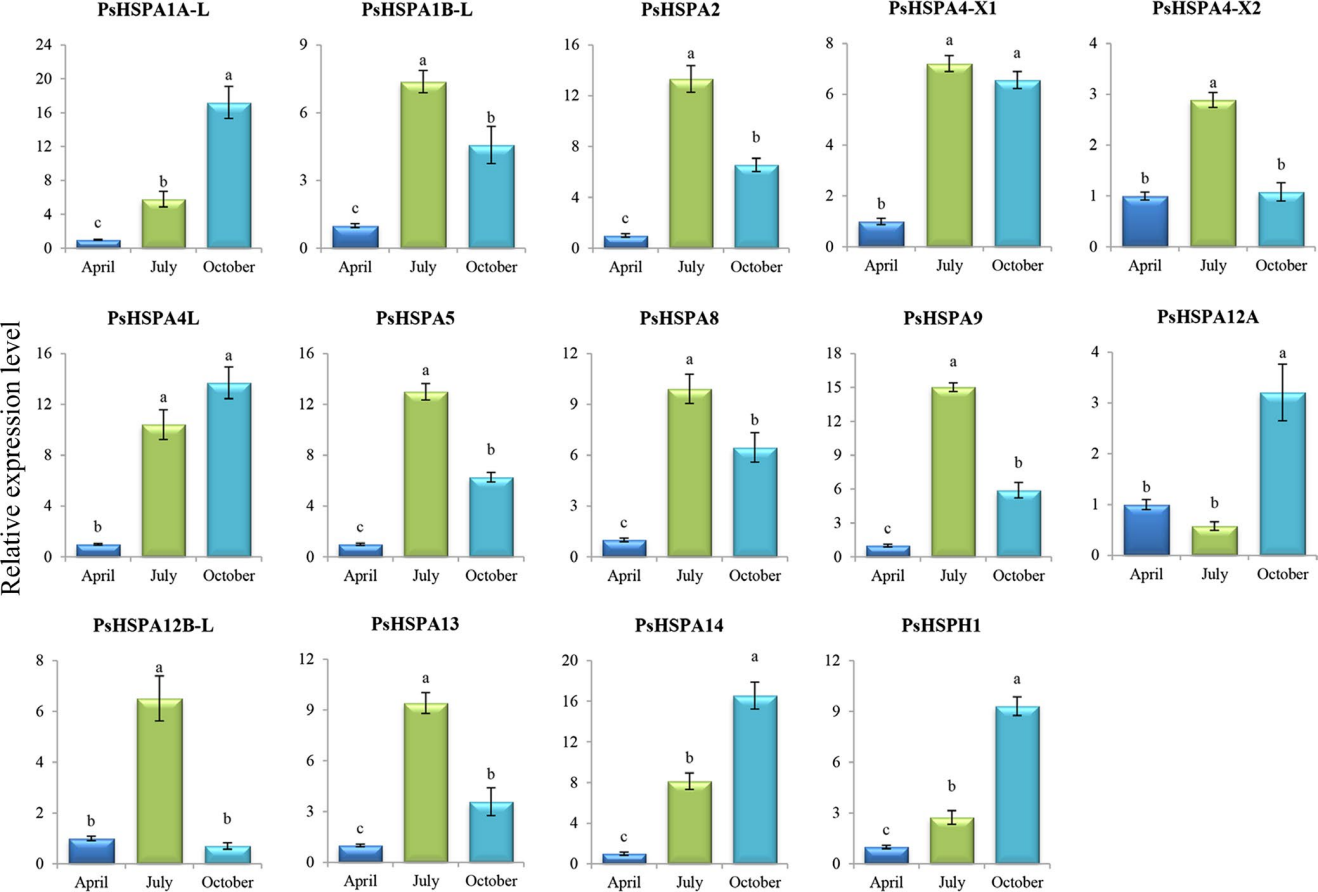
Dynamic expression of *PsHSP70/110* genes in the testis of *Pelodiscus sinensis* in April (Spring), July (Summer), and October (Autumn) determined by qRT‐PCR analysis. A different letter with a value indicates a significant difference at *p* < 0.05. Each bar shows the mean ± *SEM* of triplicate assays

The dynamic expression analyses of *PsHSP70/110* genes revealed that their expression patterns in the testis of *P. sinensis* varied with seasonal temperature changes (different months). A heat map of *PsHSP70/110* gene expression based on the FPKM values from RNA‐seq data showed that the expression abundances of different *PsHSP70/110* genes were extremely diverse in the three different *P. sinensis* libraries (Figure 4; Table 4). The *PsHSPA2* gene exhibited the highest expression level, with more than 1,500 FPKM values, whereas the *PsHSPA4‐X1*, *PsHSPA12A‐L1*, *PsHSPA12A‐L2*, *PsHSPA12A‐L3*, and *PsHSPA12A‐L4* genes were not detected in the three *P. sinensis* testis libraries. Furthermore, qRT‐PCR analysis was performed to validate the expression patterns of *PsHSP70/110* genes in April, July, and October. Comparative analysis showed that the expression tendency of most *PsHSP70/110* genes was coincident between RNA‐seq and qRT‐PCR analysis (Figure S1). The majority of *PsHSP70/110* genes were significantly differentially expressed (*p*‐value < 0.05) in July and October compared to April (Figure 5). Moreover, *PsHSPA1B‐L*, *PsHSPA2*, *PsHSPA5*, *PsHSPA8*, *PsHSPA9*, and *PsHSPA13* were significantly upregulated in July and then declined in October, indicating their temperature susceptibility. Additionally, several genes, such as *PsHSPA1A‐L*, *PsHSPA4L*, *PsHSPA12A*, *PsHSPA14*, and *PsHSPH1*, exhibited the highest expression levels in October.

**Table 4:** The FPKM values of *PsHSP70/110* gene expression in the testis of *Pelodiscus sinensis* determined by RNA‐seq analysis

Gene name	Gene ID	FPKM in MT‐1	FPKM in MT‐2	FPKM in MT‐3	Log2 (MT‐2/MT‐1)	Log2 (MT‐3/MT‐1)
PsHSPA1A‐L	ENSPSIG00000001652	6.10	2,067.89	1,575.81	8.40	8.02
PsHSPA1B‐L	ENSPSIG00000010036	14.86	1,655.57	1,172.25	6.80	6.31
PsHSPA2	ENSPSIG00000001054	2,360.52	1,815.44	2,343.12	−0.38	0.01
PsHSPA4‐X2	ENSPSIG00000013077	148.56	316.93	353.56	1.09	1.26
PsHSPA4L	ENSPSIG00000009008	456.70	846.30	943.26	0.89	1.06
PsHSPA5	ENSPSIG00000005450	470.85	285.08	474.84	−0.72	0.02
PsHSPA8	ENSPSIG00000012450	415.30	638.48	446.13	0.62	0.10
PsHSPA9	ENSPSIG00000012706	266.00	169.26	169.90	−0.65	−0.64
PsHSPA12A	ENSPSIG00000004375	89.51	113.45	83.67	0.34	−0.09
PsHSPA12B‐L	ENSPSIG00000004539	1.35	2.19	0.01	0.70	−0.70
PsHSPA13	ENSPSIG00000016198	70.71	87.39	161.55	0.31	1.20
PsHSPA14	ENSPSIG00000016234	57.48	82.66	96.88	0.52	0.76
PsHSPH1	ENSPSIG00000008548	43.49	25.27	32.48	−0.78	−0.41

### 3.5 Interaction network of PsHSP70/110 proteins

**Figure 6:**
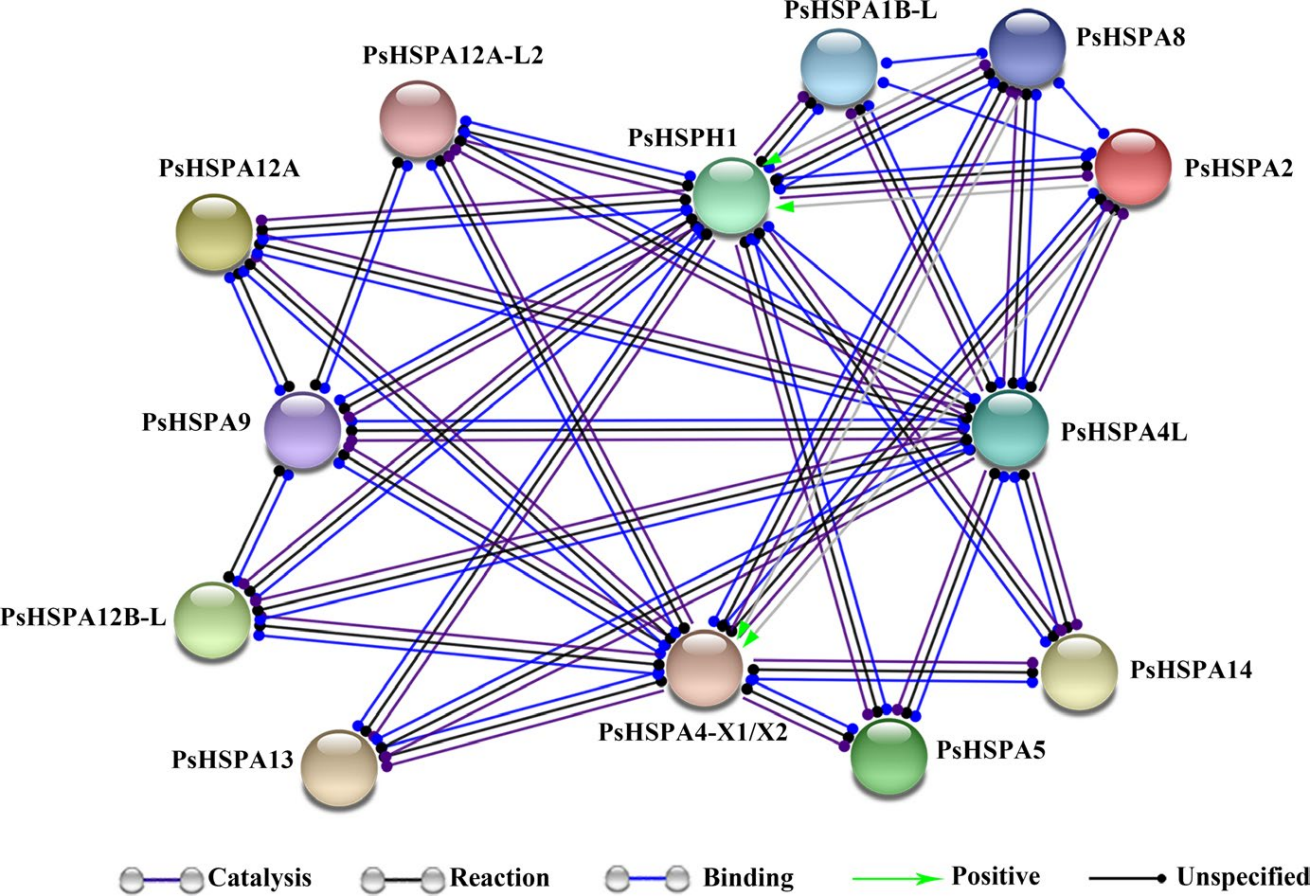
Interaction networks of PsHSP70/110 proteins. The catalysis, reaction, and binding relationships are indicated with purple, black, and blue lines, respectively. The green arrows represent the positive interaction relationship

A putative interaction network of PsHSP70/110 proteins was proposed, which involved 14 PsHSP70/110 proteins interacting with each other (Figure 6; Table S3). Four proteins (PsHSPA1A‐L, PsHSPA12A‐L1, PsHSPA12A‐L3, and PsHSPA12A‐L4) were not found in the network. The protein–protein associations showed that the majority of PsHSP70/110 proteins can interact with multiple homologous genes in *P. sinensis*. The blue, purple, and black lines represent the binding, catalysis, and reaction relationships, respectively (Figure 6). The green arrows represent positive interactions with four pairs of PsHSP70/110 proteins. Two proteins, PsHSPH1 and PsHSPA4L, were related to the most proteins, with 11 interacting genes, followed by the PsHSPA4‐X1 and PsHSPA4‐X2 proteins, with nine interacting genes. Nevertheless, PsHSPH1 and PsHSPA4L were associated with each other only by the binding line, and PsHSPA1B‐L, PsHSPA2, and PsHSPA8 exhibited the only binding relationships.

### 3.6 Functional enrichment of PsHSP70/110 proteins

The protein interaction associations among PsHSP70/110 proteins were predicted in this study and are described above. Furthermore, functional enrichment analysis of PsHSP70/110 proteins showed that five kinds of INTERPRO protein domains and features and one kind of PFAM protein domain were significantly enriched, with *p* ≤ 0.05 (Table 5). Protein domain enrichments showed that 14 interacting PsHSP70/110 proteins contained the IPR013126 and PF00012 domains, which indicate structural features of Hsp70 proteins. Moreover, the PsHSP70/110 proteins were assigned to four KEGG pathways, including “Protein processing in endoplasmic reticulum” (ID 4141, six genes), “MAPK signaling pathway” (ID 4010, three genes), “Spliceosome” (ID 3040, three genes), and “Endocytosis” (ID 4144, three genes) pathways (Table 5). Remarkably, three proteins, PsHSPA1B‐L (ENSPSIP00000011084), PsHSPA2 (ENSPSIP00000001055), and PsHSPA8 (ENSPSIP00000013985), were involved in the MAPK signaling pathway, which provides valuable information for exploring their functions in *P. sinensis*.

**Table 5:** Functional enrichment of *PsHSP70/110* genes in *Pelodiscus sinensis*

Category	Pathway ID	Pathway description	Gene count	False discovery rate	Enriched proteins
KEGG Pathways	4141	Protein processing in endoplasmic reticulum	6	1.77E−07	PsHSPA1B‐L, PsHSPA2, PsHSPA4L, PsHSPA5, PsHSPA8, PsHSPH1
	4010	MAPK signaling pathway	3	3.08E−02	PsHSPA1B‐L, PsHSPA2, PsHSPA8
	3040	Spliceosome	3	7.94E−03	PsHSPA1B‐L, PsHSPA2, PsHSPA8
	4144	Endocytosis	3	2.08E−02	PsHSPA1B‐L, PsHSPA2, PsHSPA8
INTERPRO protein domains and features	IPR013126	Heat‐shock protein 70 family	14	2.16E−35	PsHSPA1A‐L, PsHSPA1B‐L, PsHSPA2, PsHSPA4, PsHSPA4L, PsHSPA5, PsHSPA8, PsHSPA9, PsHSPA12A‐L1, PsHSPA12A‐L3, PsHSPA12A‐L4, PsHSPA13, PsHSPA14, PsHSPH1
	IPR018181	Heat‐shock protein 70, conserved site	11	4.54E−32	PsHSPA1A‐L, PsHSPA1B‐L, PsHSPA2, PsHSPA4, PsHSPA4L, PsHSPA5, PsHSPA8, PsHSPA9, PsHSPA13, PsHSPA14, PsHSPH1
	IPR029047	Heat‐shock protein 70 kD, peptide‐binding domain	10	1.01E−28	PsHSPA1A‐L, PsHSPA1B‐L, PsHSPA2, PsHSPA4, PsHSPA4L, PsHSPA5, PsHSPA8, PsHSPA9, PsHSPA14, PsHSPH1
	IPR029048	Heat‐shock protein 70 kD, C‐terminal domain	8	2.43E−21	PsHSPA1B‐L, PsHSPA2, PsHSPA4, PsHSPA4L, PsHSPA5, PsHSPA8, PsHSPA9, PsHSPH1
	IPR026685	Heat‐shock 70‐kDa protein 12A	5	3.62E−06	PsHSPA12A, PsHSPA12A‐L1, PsHSPA12A‐L2, PsHSPA12A‐L3, PsHSPA12A‐L4
PFAM protein domains	PF00012	Hsp70 protein	14	9.78E−36	PsHSPA1A‐L, PsHSPA1B‐L, PsHSPA2, PsHSPA4, PsHSPA4L, PsHSPA5, PsHSPA8, PsHSPA9, PsHSPA12A‐L1, PsHSPA12A‐L3, PsHSPA12A‐L4, PsHSPA13, PsHSPA14, PsHSPH1

### 3.7 Protein expression of PsHSPA1B‐L, PsHSPA2, and PsHSPA8 in the testis of *P. sinensis* during spermatogenesis

**Figure 7:**
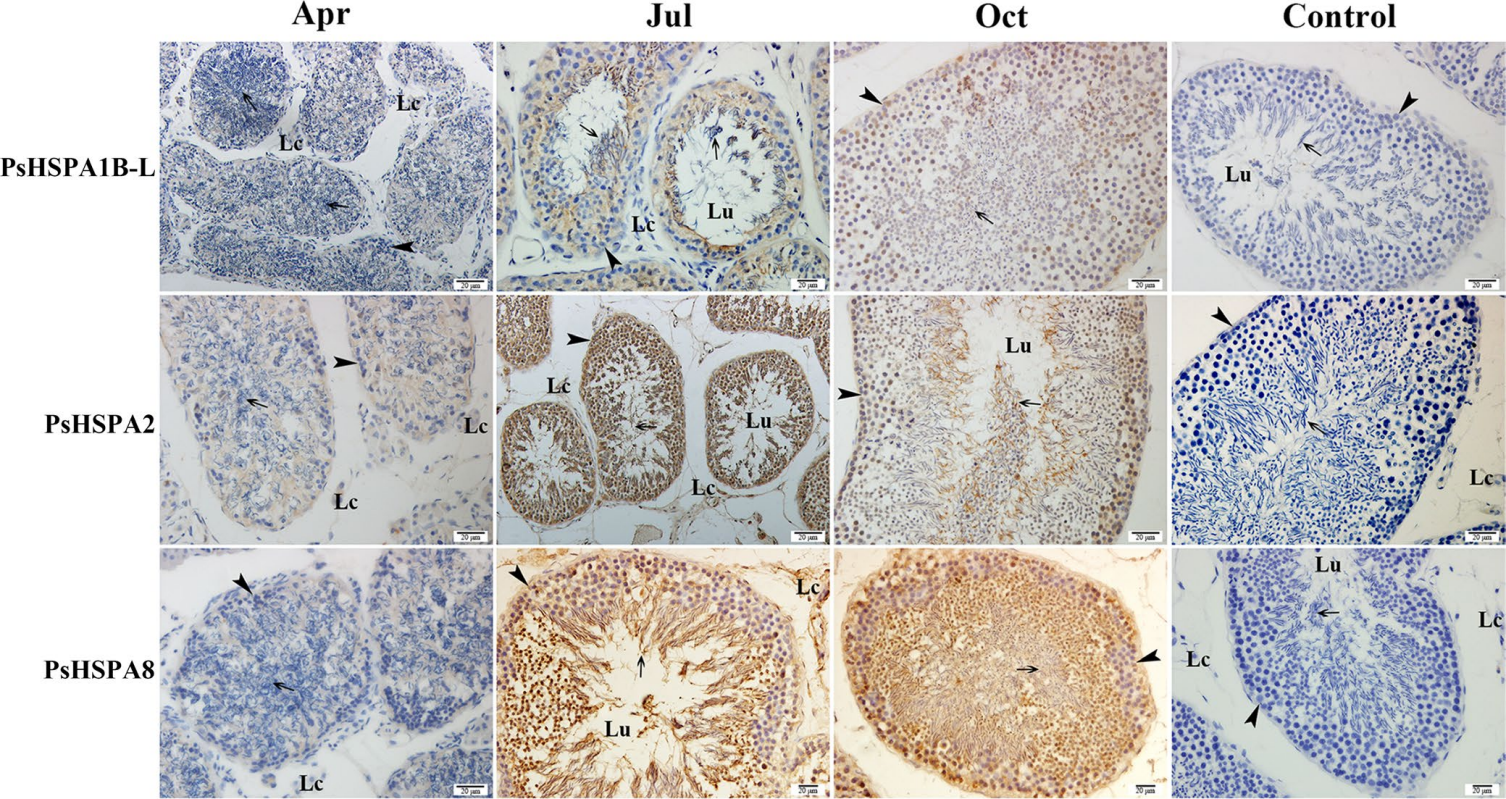
Immunostaining of PsHSPA1B‐L, PsHSPA2, and PsHSPA8 in the testis of *Pelodiscus sinensis* during spermatogenesis. Arrowhead, Spermatogonia; arrow, spermatozoa; Lc, Leydig cell; Lu, lumen. Scale bar = 20 µm

The protein expression analyses of PsHSPA1B‐L, PsHSPA2, and PsHSPA8 by IHC in different months (April, July, and October) showed that the positive reactions for the PsHSPA1B‐L, PsHSPA2, and PsHSPA8 proteins in the testis of *P. sinensis* were similar within a given month (Figure 7). Weak immunostaining of the three proteins was observed in April, whereas intense immunostaining was observed in July. In addition, the testis displayed moderate immunoreactivity of the three proteins in October. No staining was detected in the negative control sections. The results of IHC analysis were coincident with the mRNA expression variations of PsHSPA1B‐L, PsHSPA2, and PsHSPA8 determined by qRT‐PCR analysis in response to seasonal temperature changes.

### 3.8 Characterization and protein structure analysis of PsHSPA1B‐L, PsHSPA2, and PsHSPA8 proteins

**Figure 8:**
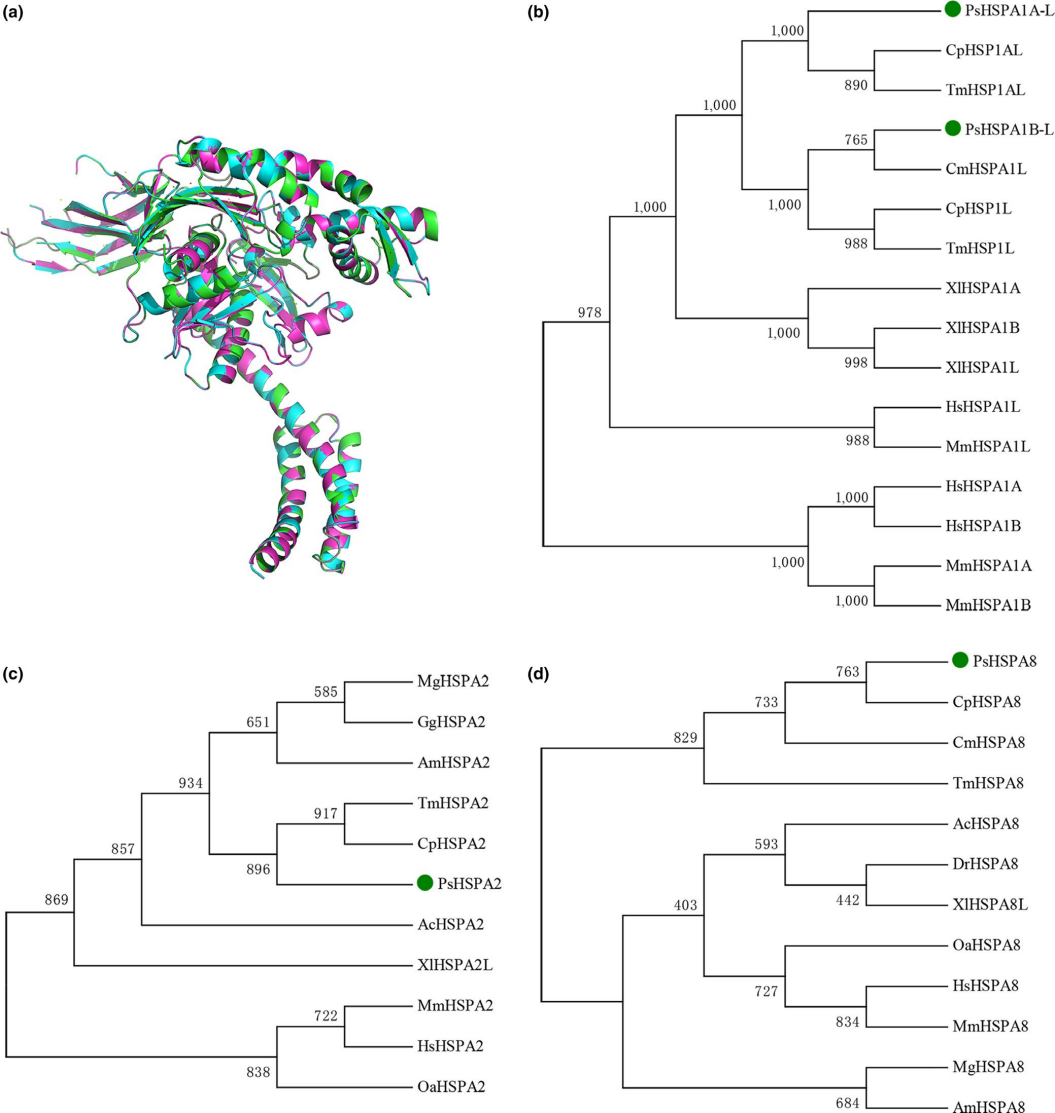
Characterization of PsHSPA1B‐L, PsHSPA2, and PsHSPA8 proteins. (a) Comparison of the predicted 3D protein structures among PsHSPA1B‐L (green), PsHSPA2 (blue), and PsHSPA8 (red). (b–d) Phylogenetic analysis of PsHSPA1B‐L, PsHSPA2, PsHSPA8 (in green circles), and their homologs from other species using bootstrap values with 1,000 replications. Ac, *Anolis carolinensis*; Am, *Alligator mississippiensis*; Cm, *Chelonia mydas*; Cp, *Chrysemys picta bellii*; Dr, *Danio rerio*; Gg, *Gallus gallus*; Hs, *Homo sapiens*; Mg, *Meleagris gallopavo*; Mm, *Mus musculus*; Oa, *Ornithorhynchus anatinus*; Tm, *Terrapene mexicana triunguis*; Xl, *Xenopus laevis*

An overview of the analysis between PsHSPA1B‐L, PsHSPA2, and PsHSPA8 proteins revealed that they exhibited high amino acid identity (Table 2) and similar conserved protein domains (Figure 1) and that they were enriched in the same KEGG pathway of MAPK signaling (Table 5). Prediction of the 3D protein structures of PsHSPA1B‐L, PsHSPA2, and PsHSPA8 showed that the three proteins presented very similar structures and binding sites, especially between PsHSPA2 and PsHSPA8 (Figure 8a; Figure S2; Table 6). More detailed phylogenetic analysis showed that PsHSPA1B‐L, PsHSPA2, and PsHSPA8 shared the closest relationships with their corresponding homologous genes from western painted turtle (*Chrysemys picta bellii*), green sea turtle (*C. mydas*), and three‐toed box turtle (*Terrapene mexicana triunguis*) (Figure 8b–d). Moreover, the mRNA and protein expression patterns of *PsHSPA1B‐L*, *PsHSPA2*, and *PsHSPA8* genes were similar in the testis of *P. sinensis* (Figure 5; Figure 7), which further validated the putative parallel functions of the three genes in certain biological processes. Furthermore, the PsAPAF1 (ENSPSIG00000011998) protein of *P. sinensis*, a potential interacting protein of PsHSP70/110 (Beere et al., 2000), was selected and subjected to interaction analysis. The predicted protein interaction revealed that PsAPAF1 was associated with the PsHSPA1B‐L, PsHSPA2, and PsHSPA8 proteins via both binding and catalysis relationships (Figure S2), which implied that *PsHSPA1B‐L*, *PsHSPA2*, and *PsHSPA8* may play similar roles and interact with the *PsAPAF1* gene in *P. sinensis*.

**Table 6:** Detailed information for 3D structure homology modeling in PsHSP70/110 proteins

Gene	Top template	PDB molecule	Confidence	Coverage	Aligned residues	Alpha helix (%)	Beta strand (%)	Predicted binding site
PsHSPA2	c3d2fC	heat‐shock protein homolog sse1	100	97	5–623	39	28	GLY13, THR14, THR15, TYR16, LYS72, GLY204, GLY205, GLY206, GLY233, GLU234, GLU271, LYS274, ARG275, SER278, GLY342, SER343, ARG345
PsHSPA8	c3d2fC	heat‐shock protein homolog sse1	100	95	4–620	39	28	GLY12, THR13, THR14, TYR15, LYS71, GLY201, GLY202, GLY203, GLY230, GLU231, GLU268, LYS271, ARG272, SER275, GLY339, SER340, ARG342
PsHSPA1B‐L	c3d2fC	heat‐shock protein homolog sse1	100	96	2–553	43	26	GLY117, GLY118, GLY119, THR120, GLY146, GLU147, ASP150, GLU184, LYS187, ARG188, SER191, GLY255, SER256, ARG258, ILE259

## 4 DISCUSSION

### 4.1 Overview of *PsHSP70/110* genes in *P. sinensis*

The central biological roles of Hsp70/110 in various biological and physiological processes are attributed to their chaperone activity and their structurally and functionally conservative properties in evolution (Lindquist & Craig, 2003). Considerable evidence has demonstrated that the protein structure and conserved domains of Hsp70/110 proteins play roles in modulating multiple cellular processes induced by a wide variety of stimuli (Gupta et al., 2010). In the present study, protein structure analysis showed that 18 PsHSP70/110 proteins contained the conserved NBD domains that are prominent structural features of Hsp70, consistent with others’ observations (20077; Song et al., 2016). Protein functional enrichments also showed that the majority of PsHSP70/110 proteins were significantly enriched in IPR013126 and PF00012 domains and were described as “Heat shock protein 70.” Additionally, the analysis of physical and chemical characteristics showed that the approximate molecular weight of most PsHSP70 family proteins was 70 kDa. These findings revealed the reliability of the identified PsHSP70/110 candidates in *P. sinensis* and supported the following studies.

### 4.2 Conservation of *PsHSP70/110* family genes in evolution

Hsp70 is the most conserved heat‐shock stress protein in evolution, and its intrinsic functions and conserved domains are responsible for its conservation across species (Kiang & Tsokos, 1998; Murphy, 2013). In this study, a genomewide search identified 18 PsHSP70/110 family members in *P. sinensis*, which is comparable to the number of Hsp70/110 genes in other species. Phylogenetic analysis and classification of Hsp70 proteins from 13 selected animals indicated that some members of this group, such as HSPA12 genes, were ubiquitous in these species. Specifically, *PsHSPA1B‐L*,* PsHSPA2*, and *PsHSPA8* showed the closest phylogenetic relationships with homologs from *C. picta bellii*, *C. mydas*, and *T. mexicana triunguis*, which is concordant with the evolutionary relationships among these species. In addition, a similar number of Hsp70 family genes were found between *P. sinensis* and other species, suggesting the evolutionary conservation of Hsp70 among different species (Song et al., 2016; Wang et al., 2019). Remarkably, HSPA2, HSPA8, and their homologous genes were assigned to the same subfamily of HSPA2/8 and exhibited the closest phylogenetic relationships, which is consistent with previous reports (Song et al., 2016). In general, comparative analysis can provide a foundation for a better understanding of the evolutionary relationships of PsHSP70/110.

In agreement with a previous report by 2016), the family of PsHSP70/110 genes examined in our studies was composed of PsHSP70 and PsHSP110 proteins. In addition to the difference of one domain between the peptide‐binding domain and the C‐terminal region, Hsp110 exhibits high homology and a similar crystal structure to Hsp70 (Polier, Dragovic, Hartl, & Bracher, 2008), although Hsp110 is a divergent Hsp70 family member. In this study, one PsHSP110 was discovered, which was named PsHSPH1 in *P. sinensis*. On the basis of its structure and sequence, PsHSP110 was included in the subfamily of PsHSP70, and these proteins were studied and discussed together. Sequence alignment showed that PsHSPH1 shared less than 40% identity with most PsHSP70 proteins. Interestingly, PsHSPH1 presented higher sequence identities with PsHSPA4L, PsHSPA4‐X1, and PsHSPA4‐X2, and these four members were classified into a closer subgroup by phylogenetic analysis, with similar conserved domains. The observations suggested that PsHSP70/110 family genes with close phylogenetic relationships and similar protein structures may present similar potential roles in *P. sinensis*.

### 4.3 Characterization of *PsHSP70/110* gene expression and putative roles

The induction and accumulation of Hsp70/110 are tightly associated with a range of environmental and physical stresses (Gupta et al., 2010; Srivastava, 2002). In this study, tissue‐specific expression analysis revealed that most *PsHSP70/110* genes were constitutively expressed in different tissues of *P. sinensis*, which suggested that *PsHSP70/110* genes may be important for organismic homeostasis. Notably, several genes, such as *PsHSPA1A‐L*, *PsHSPA1B‐L*, *PsHSPA2*, *PsHSPA5*, *PsHSPA8*, and *PsHSPA9*, exhibited specific high expression in the testis and oviduct of *P. sinensis*. Importantly, the high expression of the *PsHSPA2* gene in the *P. sinensis* testis was consistent with previous reports that showed a high level of *HSPA2* in the human testis (Daugaard et al., 2007; Son et al., 1999; Su et al., 2004), implying a potential special role of *PsHSPA2* in the germ cells of *P. sinensis*. Substantial experimental evidence has revealed that Hsp70 is one of the positive necessary factors for tumor cell survival and, on the contrary, that Hsp70 negatively modulates apoptosis (Goloudina et al., 2012; Rérole et al., 2011). In response to stressful conditions and during diverse developmental processes, apoptosis, which is essential for tissue homeostasis, is conspicuous in multicellular organisms (Meier, Finch, & Evan, 2000). In seasonally breeding species, apoptosis is responsible for testicular atrophy during seasonal reproductive regression (Young & Nelson, 2001). Moreover, apoptosis plays a critical role in seasonal spermatogenesis by eliminating defective germ cells or cells carrying DNA mutations (L^2017^; Russell, Chiarinigarcia, Korsmeyer, & Knudson, 2001). Seasonal spermatogenesis is characteristic of temperate and boreal reptilian species, including *P. sinensis* (Gribbins, 2011; Liu et al., 2017). Our previous studies examining the testis of *P. sinensis* demonstrated dynamic changes in apoptosis during spermatogenesis and showed that apoptosis was closely correlated with seasonal temperature variation: A mass of apoptotic cells was detected in April (spermatogenically quiescent phase), while decreased apoptosis was observed in July and October (intermediate and late spermatogenesis) (Liu et al., 2017). Furthermore, the constitutive or inducible expression of Hsp70 can interfere with stress‐induced apoptosis and is implicated in temperature fluctuations (Dang et al., 2015; Murphy, 2013). Studies in the teleost *Prochilodus argenteus* suggested that HSP70 may protect germ cells from apoptosis during breeding cycles, and a decrease in HSP70 expression and increase in apoptosis may facilitate testicular remodeling after the reproductive season (Domingos et al., 2013). Additionally, the targeted disruption of Hsp70 in mice leads to developmental arrest and apoptosis of spermatocytes, resulting in infertility (Dix et al., 1996). In this study, both transcriptomic and qRT‐PCR analyses showed that the dynamic expression of most *PsHSP70/110* genes was related to the temperature range. Remarkably, several genes, such as *PsHSPA1B‐L*, *PsHSPA2*, and *PsHSPA8*, were significantly upregulated in both July and October compared with April and exhibited an inverse tendency toward apoptosis during seasonal spermatogenesis, which implied that these genes may negatively regulate seasonal apoptosis in *P. sinensis*.

Furthermore, PsHSPA1B‐L, PsHSPA2, and PsHSPA8 were implicated in the MAPK signaling pathway in the present study by functional enrichment, indicating that these proteins may function through participating in MAPK signaling. MAPK plays fundamental roles in cellular stress responses and regulates apoptosis through the specific phosphorylation of apoptotic factors, such as p53 (Chang & Karin, 2001; Lee et al., 2006; Taylor, Zheng, Liu, & Thompson, 2013). Apaf1, one of the important components of the p53 signaling pathway, can mediate apoptosis through its association with procaspase‐9 (Zou, Li, Liu, & Wang, 1999). Other evidence has strongly suggested that the event of Hsp70 binding to Apaf1 seems to eliminate the oligomerization of Apaf1 and procaspase‐9 and then suppresses apoptosis (Beere & Green, 2001; Beere et al., 2000; Saleh, Srinivasula, Balkir, Robbins, & Alnemri, 2000). As expected, we found protein interactions of PsHSPA1B‐L, PsHSPA2, PsHSPA8, and PsAPAF1 with binding relationships. Moreover, IHC analysis showed that the protein expression of PsHSPA1B‐L, PsHSPA2, and PsHSPA8 exhibited significant upregulation in July and October. More importantly, a distinct decrease in apoptosis in the testis of *P. sinensis* was detected in October (MT‐3) (Liu et al., 2017). These observations indicated that the high protein levels of PsHSPA1B‐L, PsHSPA2, and PsHSPA8 as well as the interaction with PsAPAF1 may favor the inhibition of apoptosis during spermatogenesis in *P. sinensis*. In addition, in the current study, PsHSPA8, also known as PsHsc70, encoding the predominant cognate member of the PsHSP70 family, was systematically characterized in *P. sinensis* for the first time to the authors’ knowledge. Hsc70 is the cochaperone of the antiapoptotic modulator BAG1, and BAG1 can bind to its ATPase domains to modulate chaperone activities and influence apoptotic responses (Song, Takeda, & Morimoto, 2001; Stuart et al., 1998). Taken together, these findings reveal the potential functions of critical PsHSP70/110 proteins in regulating apoptosis and associated with spermatogenesis in *P. sinensis*.

## 5 CONCLUSION

In this study, a total of 18 *PsHSP70/110* family genes were identified and comprehensively analyzed in *P. sinensis*. All the PsHSP70/110 proteins contained the conserved NBD domain, which represents a typical structural feature of Hsp70. Classification and phylogenetic analysis of PsHSP70/110 and their homologs demonstrated that Hsp70/110 was conserved in evolution across various species. Moreover, the expression profiling of these *PsHSP70/110* genes varied considerably in different tissues of *P. sinensis*. Notably, several genes, such as *PsHSPA1B‐L*, *PsHSPA2*, and *PsHSPA8*, were significantly differentially expressed in the testis of *P. sinensis* in different months. Functional enrichments showed that PsHSPA1B‐L, PsHSPA2, and PsHSPA8, with putative interaction relationships, were involved in the MAPK signaling pathway. Further analysis indicated that the high protein expression of PsHSPA1B‐L, PsHSPA2, and PsHSPA8 and their binding to PsAPAF1 may account for the decrease in apoptosis during spermatogenesis in *P. sinensis*, which suggested a putative vital role of critical PsHSP70/110 in regulating apoptosis. The findings of this study will provide insights into the potential functional roles of PsHSP70/110 in *P. sinensis*.

## CONFLICTS OF INTEREST

The authors declare that they have no competing interests.

## AUTHOR CONTRIBUTIONS

The authors have made the following declarations about their contributions: TL and HZ conceived and designed the study. TL, YH, and YL analyzed the data and performed the experiments. TL wrote the manuscript. TL and HZ revised the manuscript. All authors read and approved the final manuscript.

## Data Availability

Transcriptomic data: NCBI SRA: SRX2351846 (MT‐1), SRX2352135 (MT‐2), and SRX2352136 (MT‐3).

## References

[ece35264-bib-0001] Arya, R. , Mallik, M. , & Lakhotia, S. C. (2007). Heat shock genes‐integrating cell survival and death. Journal of Biosciences, 32, 595–610 10.1007/s12038-007-0059-3 17536179

[ece35264-bib-0002] Beere , H. M. , & Green , D. R. (2001). Stress management‐heat shock protein‐70 and the regulation of apoptosis. Trends in Cell Biology, 11, 6–10. 10.1016/S0962-8924(00)01874-2 11146277

[ece35264-bib-0003] Beere, H. M. , Wolf, B. B. , Cain, K. , Mosser, D. D. , Mahboubi, A. , Kuwana, T. , & Green, D. R. (2000). Heat‐shock protein 70 inhibits apoptosis by preventing recruitment of procaspase‐9 to the Apaf‐1 apoptosome. Nature Cell Biology, 2, 469–475. 10.1038/35019501 10934466

[ece35264-bib-0004] Bertelsen, E. B. , Chang, L. , Gestwicki, J. E. , & Zuiderweg, E. R. P. (2009). Solution conformation of wild‐type E. coli Hsp70 (DnaK) chaperone complexed with ADP and substrate. Proceedings of the National Academy of Sciences of the United States of America, 106, 8471–8476. 10.1073/pnas.0903503106 19439666 PMC2689011

[ece35264-bib-0005] Chang, L. , & Karin, M. (2001). Mammalian MAP kinase signalling cascades. Nature, 410, 37–40. 10.1038/35065000 11242034

[ece35264-bib-0006] Chen, S. , Zhang, L. , Le, Y. , Waqas, Y. , Chen, W. , Zhang, Q. , Yang, P. (2015). Sperm storage and spermatozoa interaction with epithelial cells in oviduct of Chinese soft‐shelled turtle, *Pelodiscus sinensis* . Ecology and Evolution, 5, 3023–3030. 10.1002/ece3.1575 26357535 PMC4559046

[ece35264-bib-0007] Dang, W. , Lu, H. , Gao, Y. , Xu, N. , Qu, T. , & Liu, Y. (2015). Molecular analysis of inducible Heat shock protein 70 of *Pelodiscus sinensis*, and its effects during pathogen (*Aeromonas hydrophila*) infection. Aquaculture, 442, 93–99. 10.1016/j.aquaculture.2015.02.030

[ece35264-bib-0008] Daugaard, M. , Rohde, M. , & Jäättelä, M. (2007). The heat shock protein 70 family: Highly homologous proteins with overlapping and distinct functions. FEBS Letters, 581, 3702–3710. 10.1016/j.febslet.2007.05.039 17544402

[ece35264-bib-0009] de Hoon, M. , Imoto, S. , Nolan, J. , & Miyano, S. (2004). Open source clustering software. Bioinformatics, 20, 1453–1454. 10.1093/bioinformatics/bth078 14871861

[ece35264-bib-0010] Dix, D. J. , Allen, J. W. , Collins, B. W. , Mori, C. , Nakamura, N. , Poorman‐Allen, P. , … Eddy, E. M. (1996). Targeted gene disruption of Hsp70‐2 results in failed meiosis, germ cell apoptosis, and male infertility. Proceedings of the National Academy of Sciences of the United States of America, 193, 3264‐3268. 10.1073/pnas.93.8.3264 PMC39594 8622925

[ece35264-bib-0011] Domingos, F. F. T. , Thomé, R. G. , Martinelli, P. M. , Sato, Y. , Bazzoli, N. , & Rizzo, E. (2013). Role of HSP70 in the regulation of the testicular apoptosis in a seasonal breeding teleost *Prochilodus argenteus* from the São Francisco river, Brazil. Microscopy Research and Technique, 76, 350–356. 10.1002/jemt.22173 23362090

[ece35264-bib-0012] Dragovic, Z. , Broadley, S. A. , Shomura, Y. , Bracher, A. , & Hartl, F. U. (2006). Molecular chaperones of the Hsp110 family act as nucleotide exchange factors of Hsp70s. Embo Journal, 25, 2519–2528. 10.1038/sj.emboj.7601138 16688212 PMC1478182

[ece35264-bib-0013] Gao, J. , Zhang, W. , Dang, W. , Mou, Y. , Gao, Y. , Sun, B. J. , & Du, W. G. (2014). Heat shock protein expression enhances heat tolerance of reptile embryos. Proceedings Biological Sciences, 281, 20141135 10.1098/rspb.2014.1135 25080340 PMC4132679

[ece35264-bib-0014] Goloudina, A. R. , Demidov, O. N. , & Garrido, C. (2012). Inhibition of HSP70: A challenging anti‐cancer strategy. Cancer Letters, 325, 117–124. 10.1016/j.canlet.2012.06.003 22750096

[ece35264-bib-0015] Gribbins, K. M. (2011). Reptilian spermatogenesis: A histological and ultrastructural perspective. Spermatogenesis, 1, 250–269. 10.4161/spmg.1.3.18092 22319673 PMC3271667

[ece35264-bib-0016] Guindon, S. , Dufayard, J. F. , Lefort, V. , Anisimova, M. , Hordijk, W. , & Gascuel, O. (2010). New Algorithms and methods to estimate maximum‐likelihood phylogenies: Assessing the performance of PhyML 3.0. Systematic Biology, 59, 307–321. 10.2307/25677586 20525638

[ece35264-bib-0017] Gupta, S. C. , Sharma, A. , Mishra, M. , Mishra, R. K. , & Chowdhuri, D. K. (2010). Heat shock proteins in toxicology: How close and how far? Life Science, 86, 377–384. 10.1016/j.lfs.2009.12.015 20060844

[ece35264-bib-0018] Jäättelä, M. (1995). Over‐expression of hsp70 confers tumorigenicity to mouse fibrosarcoma cells. International Journal of Cancer, 60, 689–693. 10.1002/ijc.2910600520 7860144

[ece35264-bib-0019] Jesper, N. , Mads, G. H. , Agnieszka, D. , Nicole, F. , Ulrik, L. , Maria, H. H. , … Jäättelä , M. (2004). Heat shock protein 70 promotes cell survival by inhibiting lysosomal membrane permeabilization. Journal of Experimental Medicine, 200, 425–435. 10.1084/jem.20040531 15314073 PMC2211935

[ece35264-bib-0020] Jiang, B. , Liang, P. , Deng, G. , Tu, Z. , Liu, M. , & Xiao, X. (2011). Increased stability of Bcl‐2 in HSP70‐mediated protection against apoptosis induced by oxidative stress. Cell Stress and Chaperones, 16, 143–152. 10.1007/s12192-010-0226-6 20890773 PMC3059790

[ece35264-bib-0021] Kelley, L. A. , Mezulis, S. , Yates, C. M. , Wass, M. N. , & Sternberg, M. J. (2015). The Phyre2 web portal for protein modeling, prediction and analysis. Nature Protocols, 10, 845–858. 10.1038/nprot.2015.053 25950237 PMC5298202

[ece35264-bib-0022] Kiang, J. G. , & Tsokos, G. C. (1998). Heat Shock Protein 70 kDa: Molecular biology, biochemistry, and physiology. Pharmacology and Therapeutics, 80, 183–201. 10.1016/S0163-7258(98)00028-X 9839771

[ece35264-bib-0023] Kotoglou, P. , Kalaitzakis, A. , Vezyraki, P. , Tzavaras, T. , Michalis, L. K. , Dantzer, F. , … Angelidis, C. (2009). Hsp70 Translocates to the Nuclei and Nucleoli, Binds to XRCC1 and PARP‐1, and Protects HeLa Cells from Single‐Strand DNA Breaks. Cell Stress and Chaperones, 14, 391–406. https://doi:10.1007/s12192-008-0093-6. 19089598 10.1007/s12192-008-0093-6 PMC2728274

[ece35264-bib-0024] Lee, E. R. , Kim, J. Y. , Kang, Y. J. , Ahn, J. Y. , Kim, J. H. , Kim, B. W. , Cho, S. G. (2006). Interplay between PI3K/Akt and MAPK signaling pathways in DNA‐damaging drug‐induced apoptosis. Biochimica Et Biophysica Acta, 1763, 958–968. 10.1016/j.bbamcr.2006.06.006 16905201

[ece35264-bib-0025] Lindquist, S. , & Craig, E. A. (2003). The heat‐shock proteins. Annual Review of Genetics, 22, 631–677. 10.1146/annurev.ge.22.120188.003215 2853609

[ece35264-bib-0026] Liu, T. , Chu, X. , Huang, Y. , Yang, P. , Li, Q. , Hu, L. , Chen, Q. (2016). Androgen‐related sperm storage in oviduct of Chinese Soft‐Shelled Turtle in vivo during annual cycle. Scientific Reports, 6, 20456 10.1038/srep20456. 26847578 PMC4742787

[ece35264-bib-0027] Liu, T. , Wang, L. , Chen, H. , Huang, Y. , Yang, P. , Nisar, A. , Chen, Q. (2017). Molecular and cellular mechanisms of apoptosis during dissociated spermatogenesis. Frontiers in Physiology, 8, 188 10.3389/fphys.2017.00188 28424629 PMC5372796

[ece35264-bib-0028] Liu, T. , Yang, P. , Chen, H. , Huang, Y. , Liu, Y. , Waqas, Y. , Chen, Q. (2016). Global analysis of differential gene expression related to long‐term sperm storage in oviduct of Chinese soft‐shelled turtle *Pelodiscus sinensis* . Scientific Reports, 6, 33296 10.1038/srep33296. 27628424 PMC5024102

[ece35264-bib-0029] Livak, K. J. , & Schmittgen, T. D. (2001). Analysis of relative gene expression data using real‐time quantitative PCR and the 2^−ΔΔ^ *^C^* ^T^ method. Methods, 25, 402–408. 10.1006/meth.2001.1262 11846609

[ece35264-bib-0030] Luan, W. , Li, F. , Zhang, J. , Wen, R. , Li, Y. , & Xiang, J. (2010). Identification of a novel inducible cytosolic Hsp70 gene in Chinese shrimp *Fenneropenaeus chinensis* and comparison of its expression with the cognate Hsc70 under different stresses. Cell Stress and Chaperones, 15, 83–93. https://doi:10.1007/s12192-009-0124-y. 19496024 10.1007/s12192-009-0124-y PMC2866979

[ece35264-bib-0031] Meier, P. , Finch, A. , & Evan, G. (2000). Apoptosis in development. Nature, 407, 796–801. 10.1038/35037734. 11048731

[ece35264-bib-0032] Mortazavi, A. , Williams, B. A. , McCue, K. , Schaeffer, L. , & Wold, B. (2008). Mapping and quantifying mammalian transcriptomes by RNA‐Seq. Nature Methods, 5, 621–628. 10.1038/nmeth.1226 18516045 PMC13303166

[ece35264-bib-0033] Murphy, M. E. (2013). The HSP70 family and cancer. Carcinogenesis, 34, 1181–1188. 10.1093/carcin/bgt111 23563090 PMC3670260

[ece35264-bib-0034] Park, H. S. , Cho, S. G. , Kim, C. K. , Hwang, H. S. , Noh, K. T. , Kim, M. S. , Choi, E. J. (2002). Heat shock protein hsp72 is a negative regulator of apoptosis signal‐regulating kinase 1. Molecular and Cellular Biology, 22, 7721–7730. 10.1128/MCB.22.22.7721-7730.2002 12391142 PMC134722

[ece35264-bib-0035] Park, H. S. , Lee, J. S. , Huh, S. H. , Seo, J. S. , & Choi, E. J. (2001). Hsp72 functions as a natural inhibitory protein of c‐Jun N‐terminal kinase. Embo Journal, 20, 446–456. 10.1093/emboj/20.3.446 11157751 PMC133486

[ece35264-bib-0036] Polier, S. , Dragovic, Z. , Hartl, F. U. , & Bracher, A. (2008). Structural basis for the cooperation of Hsp70 and Hsp110 chaperones in protein folding. Cell, 133, 1068–1079. 10.1016/j.cell.2008.05.022 18555782

[ece35264-bib-0037] Ravagnan, L. , Gurbuxani, S. , Susin, S. A. , Maisse, C. , Daugas, E. , & Zamzami, N. … Kroemer, G. (2001). Heat‐shock protein 70 antagonizes apoptosis‐inducing factor. Nature Cell Biology, 3, 839–843. 10.1038/ncb0901-839 11533664

[ece35264-bib-0038] Rérole, A. L. , Gobbo, J. , De, T. A. , Schmitt, E. , Jp, P. D. B. , Hammann, A. , Garrido, C. (2011). Peptides and aptamers targeting HSP70: A novel approach for anticancer chemotherapy. Cancer Research, 71, 484–495. 10.1158/0008-5472.CAN-10-1443 21224349

[ece35264-bib-0039] Russell, L. D. , Chiarinigarcia, H. , Korsmeyer, S. J. , & Knudson, C. M. (2001). Bax‐dependent spermatogonia apoptosis is required for testicular development and spermatogenesis. Biology of Reproduction, 66, 950–958. 10.1095/biolreprod66.4.950 11906913

[ece35264-bib-0040] Sabirzhanov, B. , Stoica, B. A. , Hanscom, M. , Piao, C. S. , & Faden, A. I. (2012). Over‐expression of HSP70 attenuates caspase‐dependent and caspase‐independent pathways and inhibits neuronal apoptosis. Journal of Neurochemistry, 123, 542–554. 10.1111/j.1471-4159.2012.07927.x 22909049 PMC3753080

[ece35264-bib-0041] Saldanha, A. J. (2004). Java Treeview‐extensible visualization of microarray data. Bioinformatics, 20, 3246–3248. 10.1093/bioinformatics/bth349 15180930

[ece35264-bib-0042] Saleh, A. , Srinivasula, S. M. , Balkir, L. , Robbins, P. D. , & Alnemri, E. S. (2000). Negative regulation of the Apaf‐1 apoptosome by Hsp70. Nature Cell Biology, 2, 476–483. 10.1038/35019510 10934467

[ece35264-bib-0043] Simoncelli, F. , Morosi, L. , Rosa, I. D. , Pascolini, R. , & Fagotti, A. (2010). Molecular characterization and expression of a heat‐shock cognate 70 (Hsc70) and a heat‐shock protein 70 (Hsp70) cDNAs in Rana (Pelophylax) lessonae embryos. Comparative Biochemistry and Physiology‐Part A: Molecular and Integrative Physiology, 156, 552–560. 10.1016/j.cbpa.2010.04.016 20435156

[ece35264-bib-0044] Son, W. Y. , Hwang, S. H. , Han, C. T. , Lee, J. H. , Kim, S. , & Kim, Y. C. (1999). Specific expression of heat shock protein HspA2 in human male germ cells. Molecular Human Reproduction, 5, 1122–1126. 10.1093/molehr/5.12.1122 10587366

[ece35264-bib-0045] Song, J. , Takeda, M. , & Morimoto, R. I. (2001). Bag1‐Hsp70 mediates a physiological stress signalling pathway that regulates Raf‐1/ERK and cell growth. Nature Cell Biology, 3, 276–282. 10.1038/35060068. 11231577

[ece35264-bib-0046] Song, L. , Li, C. , Xie, Y. , Liu, S. , Zhang, J. , Yao, J. , Liu, Z. (2016). Genome‐wide identification of Hsp70 genes in channel catfish and their regulated expression after bacterial infection. Fish and Shellfish Immunology, 49, 154–162. 10.1016/j.fsi.2015.12.009 26693666

[ece35264-bib-0047] Srivastava, P. (2002). Roles of heat‐shock proteins in innate and adaptive immunity. Nature Reviews Immunology, 2, 185–194. 10.1038/nri749 11913069

[ece35264-bib-0048] Stankiewicz, A. R. , Lachapelle, G. , Foo, C. P. , Radicioni, S. M. , & Mosser, D. D. (2005). Hsp70 inhibits heat‐induced apoptosis upstream of mitochondria by preventing Bax translocation. The Journal of Biological Chemistry, 280, 38729–38739. 10.1074/jbc.M509497200 16172114

[ece35264-bib-0049] Stuart, J. K. , Myszka, D. G. , Joss, L. , Mitchell, R. , McDonald, S. M. , Xie, Z. , Ely, K. R. (1998). Characterization of interactions between the anti‐apoptotic protein BAG‐1 and Hsc70 molecular chaperones. The Journal of Biological Chemistry, 273, 22506–22514. 10.1074/jbc.273.35.22506 9712876

[ece35264-bib-0050] Su, A. I. , Wiltshire, T. , Batalov, S. , Lapp, H. , Ching, K. A. , Block, D. , Hogenesch, J. B. (2004). A gene atlas of the mouse and human protein‐encoding transcriptomes. Proceedings of the National Academy of Sciences of the United States of America, 101, 6062–6067. 10.1073/pnas.0400782101 15075390 PMC395923

[ece35264-bib-0051] Taylor, C. A. , Zheng, Q. , Liu, Z. , & Thompson, J. E. (2013). Role of p38 and JNK MAPK signaling pathways and tumor suppressor p53 on induction of apoptosis in response to Ad‐eIF5A1 in A549 lung cancer cells. Molecular Cancer, 12, 35 10.1186/1476-4598-12-35 23638878 PMC3660295

[ece35264-bib-0052] Wang, X. , Wang, C. , Ban, F. , Zhu, D. , Liu, S. , & Wang, X. (2019). Genome‐wide identification and characterization of HSP gene superfamily in whitefly (*Bemisia tabaci*) and expression profiling analysis under temperature stress. Insect Science, 26, 44–57. 10.1111/1744-7917.12505 28714602

[ece35264-bib-0053] Wang, Z. , Pascualanaya, J. , Zadissa, A. , Li, W. , Niimura, Y. , Huang, Z. , Irie, N. (2013). The draft genomes of soft‐shell turtle and green sea turtle yield insights into the development and evolution of the turtle‐specific body plan. Nature Genetics, 45, 701–706. 10.1038/ng.2615 23624526 PMC4000948

[ece35264-bib-0054] Wang, Z. , Wu, Z. , Jian, J. , & Lu, Y. (2009). Cloning and expression of heat shock protein 70 gene in the haemocytes of pearl oyster (*Pinctada fucata*, Gould 1850) responding to bacterial challenge. Fish and Shellfish Immunology, 26, 639–645. 10.1016/j.fsi.2008.10.011 19026750

[ece35264-bib-0055] Wass, M. N. , Kelley, L. A. , & Sternberg, M. J. (2010). 3DLigandSite: Predicting ligand‐binding sites using similar structures. Nucleic Acids Research, 38, W469–473. 10.1093/nar/gkq406 20513649 PMC2896164

[ece35264-bib-0056] Young, K. A. , & Nelson, R. J. (2001). Mediation of seasonal testicular regression by apoptosis. Reproduction, 122, 677–685. 10.1530/rep.0.1220677 11690527

[ece35264-bib-0057] Zhang, L. , Fok, J. J. , Mirabella, F. , Aronson, L. I. , Fryer, R. A. , Workman, P. , Davies, F. E. (2013). Hsp70 inhibition induces myeloma cell death via the intracellular accumulation of immunoglobulin and the generation of proteotoxic stress. Cancer Letters, 339, 49–59. 10.1016/j.canlet.2013.07.023 23887058 PMC3778988

[ece35264-bib-0058] Zimmerman, L. M. , Vogel, L. A. , & Bowden, R. M. (2010). Understanding the vertebrate immune system: Insights from the reptilian perspective. The Journal of Experimental Biology, 213, 661–671. 10.1242/jeb.038315 20154181

[ece35264-bib-0059] Zou, H. , Li, Y. , Liu, X. , & Wang, X. (1999). An APAF‐1. cytochrome c multimeric complex is a functional apoptosome that activates procaspase‐9. The Journal of Biological Chemistry, 274, 11549–11556. 10.1074/jbc.274.17.11549 10206961

