# Supplementary figures and images for: Genomewide identification and analysis of heat‐shock proteins 70/110 to reveal their potential functions in Chinese soft‐shelled turtle Pelodiscus sinensis
Source: Ecol Evol. 2019 May 20;9(12):6968–85. doi: 10.1002/ece3.5264 (PMC6712388; doi:10.1002/ece3.5264)

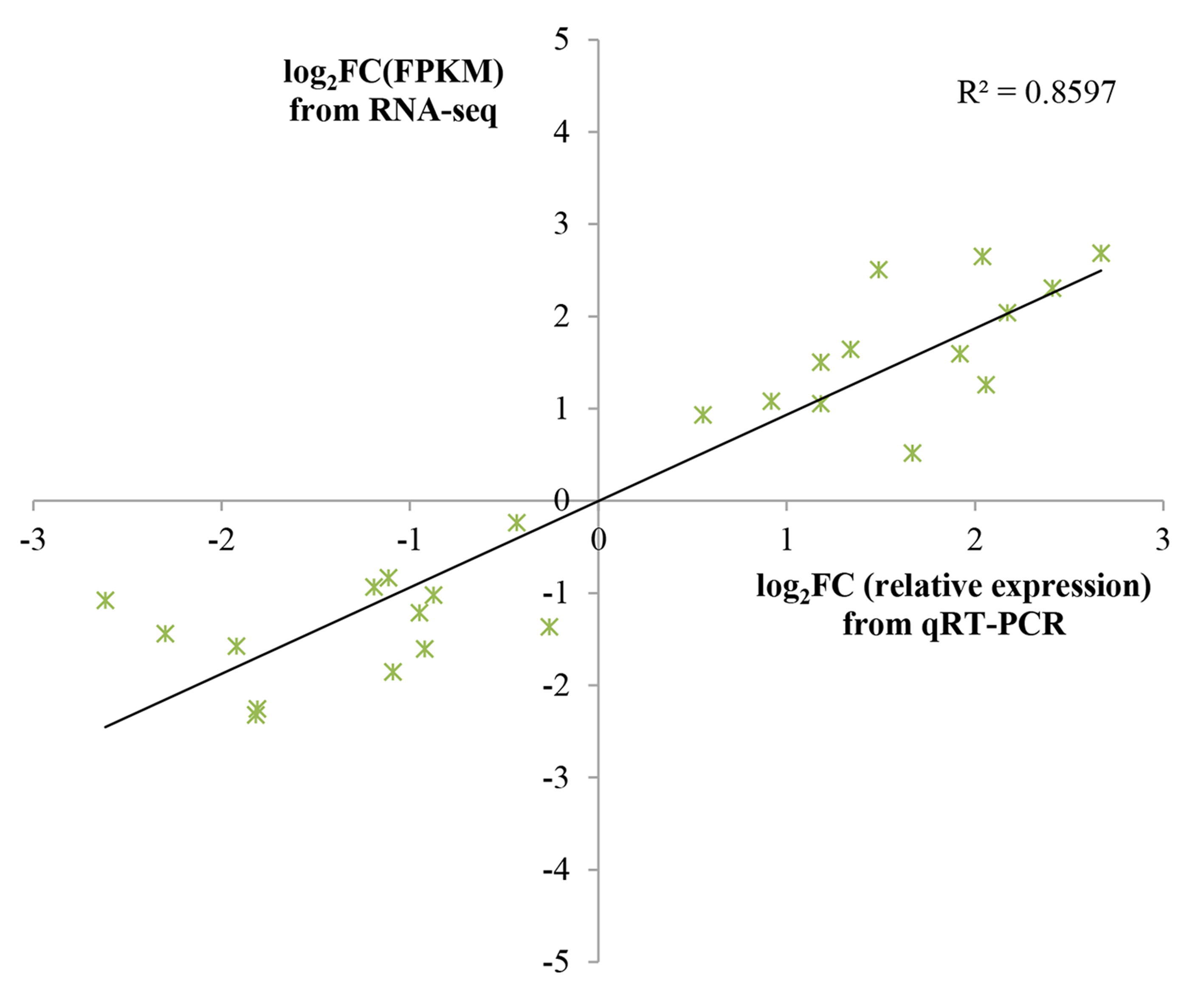

Supplement: Supplementary file 1 [file ECE3-9-6968-s001.tif]

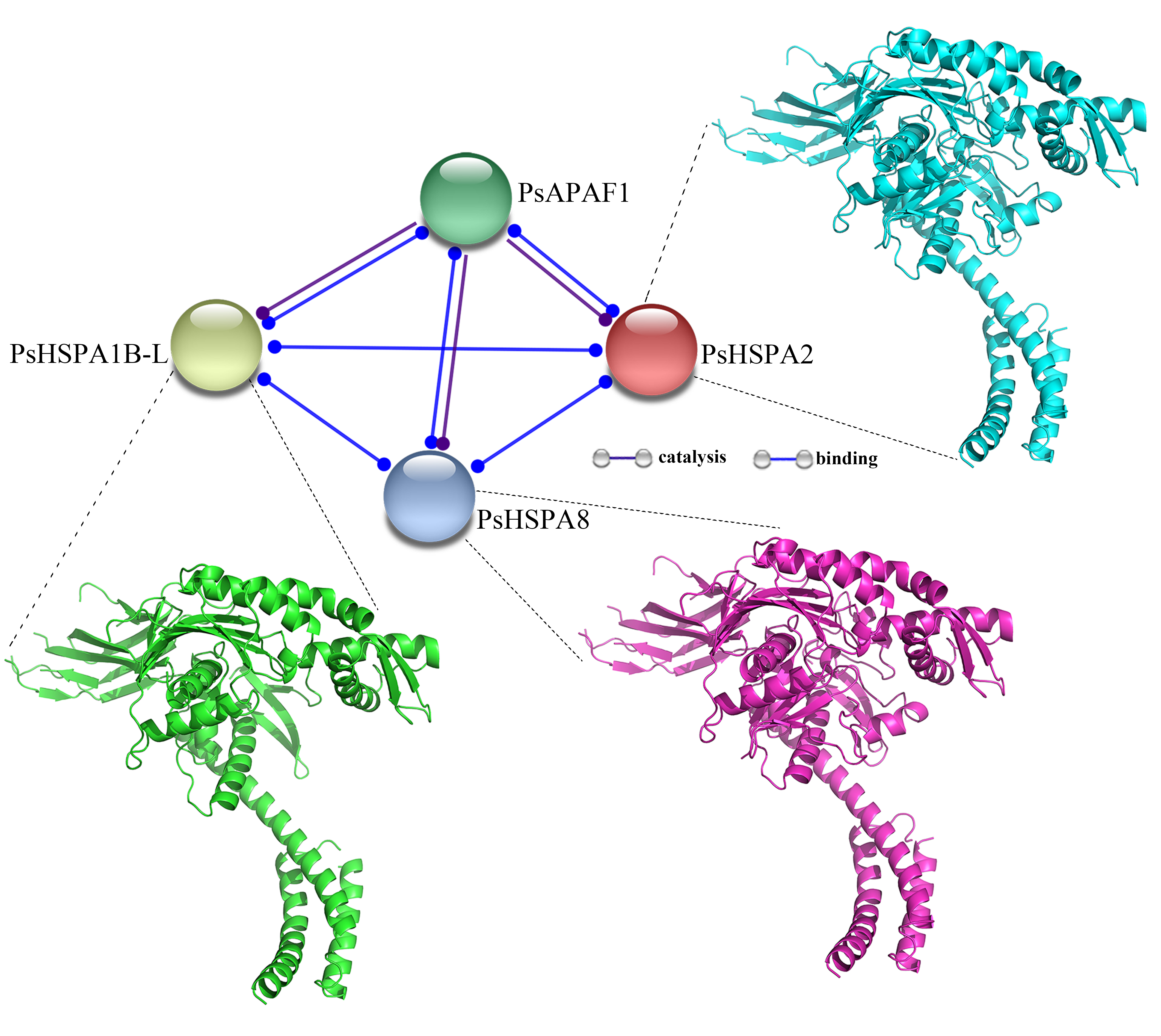

Supplement: Supplementary file 2 [file ECE3-9-6968-s002.tif]
